# Multidimensional Hybrid Metal Phosphonate Coordination Networks as Synergistic Anticorrosion Coatings

**DOI:** 10.1021/acs.inorgchem.4c02545

**Published:** 2024-08-12

**Authors:** Apostolos Fanourgiakis, Elpiniki Chachlaki, Nicoleta Plesu, Duane Choquesillo-Lazarte, Alexander M. Kirillov, Konstantinos D. Demadis

**Affiliations:** † Crystal Engineering, Growth and Design Laboratory, Department of Chemistry, University of Crete, Voutes Campus, Heraklion, Crete GR-71003, Greece; ‡ "Coriolan Drăgulescu" Institute of Chemistry, 300223 Timisoara, Romania; § Laboratorio de Estudios Cristalográficos, IACT, CSIC-Universidad de Granada, Granada 18100, Spain; ∥ Centro de Química Estrutural, Institute of Molecular Sciences, Departamento de Engenharia Química, Instituto Superior Técnico, Universidade de Lisboa, Av. Rovisco Pais, 1049-001 Lisbon, Portugal

## Abstract

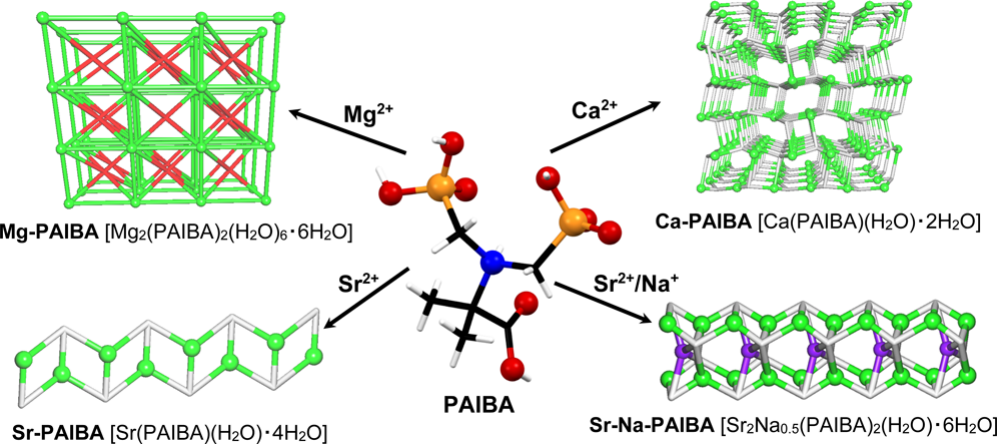

In the technologically important field of anticorrosion coatings, it is imperative to form well-defined and characterized films to protect the metal surface from corrosion. Phosphonate-based corrosion mitigation approaches are currently being exploited. Herein, the synergistic action of alkaline-earth metal ions and two carboxy-diphosphonates, PAIBA [*N,N*-bis(phosphonomethyl)-2-aminoisobutyric acid] and BPMGLY [*N,N*-*bis*(phosphonomethyl)glycine], is explored. Also, a family of four novel hybrid metal phosphonate materials is reported, Mg-PAIBA, Ca-PAIBA, Sr-PAIBA, and Sr-Na-PAIBA, whose topological analysis revealed a variety of underlying networks with the 6,10T9, **unc**, SP 1-periodic net (4,4)(0,2), and unique topologies. The synergistic metal/carboxy-diphosphonate blends were tested for their anticorrosion performance on carbon steel at preselected concentrations (0.1–1.0 mM) and pH values (4.0–6.0). The results showed an enhanced inhibitory performance in the presence of metal cations at higher concentrations. The inhibition of corrosion at pH 5.0 in the presence of BPMGLY, PAIBA, and their combination with Sr^2+^ was investigated in detail using electrochemical measurements. Enhanced inhibition was achieved with a 1:1 Sr^2+^/BPMGLY (or PAIBA) binary system. Polarization curves indicated that the system is a “mixed” inhibitor. This study widens the family of carboxyphosphonate coordination polymers, showing their potential as attractive hybrid coatings with anticorrosion performance.

## Abstract 2

The coordination chemistry of *N,N*-bis(phosphonomethyl)-2-aminoisobutyric acid (PAIBA) is explored with the alkaline-earth metal ions Mg, Ca, and Sr. Synergistic blends of PAIBA with Mg^2+^, Ca^2+^, Sr^2+^, and Ba^2+^ show effective anticorrosion protection of steel surfaces. The Sr^2+^/PAIBA system was further studied with electrochemical techniques and compared to the related Sr^2+^/BPMGLY (*N,N*-*bis*(phosphonomethyl)glycine) system.

## Introduction

The growth of metal phosphonate chemistry has produced a wide variety of well-characterized hybrid inorganic–organic compounds with a plethora of structural architectures. Exploitation of their functionality has led to several potential applications, such as gas storage,^1^ ion exchange,^2^ catalysis,^3^ intercalation,^4^ proton conductivity,^5^ electrical conductivity,^6^ optics,^7^ and protective coatings,^8^ just to mention a few. Building upon the latter, we have been investigating the formation and functionality of metal phosphonate hybrid coatings for the industrially important protection of steel surfaces from corrosion.^9^

The field of metal phosphonate hybrid coatings has expanded in the last decades, mainly due to the discovery and synthetic development of new phosphonate molecules.^8,9^ Such systems take advantage of the metal ions commonly found in natural waters (mostly alkaline-earth cations) or Zn^2+^ (which is purposely added to augment corrosion protection due to surface-formed Zn(OH)_2_).^10^ The chemical identity of an anticorrosion hybrid coating has a direct impact on its effectiveness. Intrinsic factors that play a significant role include the nature of the metal ion (charge, ionic radius, harness), the structure of the phosphonate molecule (number of phosphonate groups, hydrophobicity/hydrophilicity balance, presence of other binding moieties), the chemical structure, and the crystal packing of the actual coating. Extrinsic factors include system temperature, solution pH, type of metallurgy, flow rate, and presence of aggravating ions, e.g., chlorides. Systematic studies were published, including a wide spectrum of phosphonate additives, such as triphosphonates (Zn^2+^/amino-tris(methylenephosphonate) blends),^11^ tetra-phosphonates (Zn^2+^/hexamethylenediamine-*tetrakis*(methylenephosphonate) blends),^12^ and “mixed” carboxy/phosphonates (Ca^2+^/Sr^2+^/Ba^2+^/hydroxyphosphonoacetate).^13^

**Figure 1:**
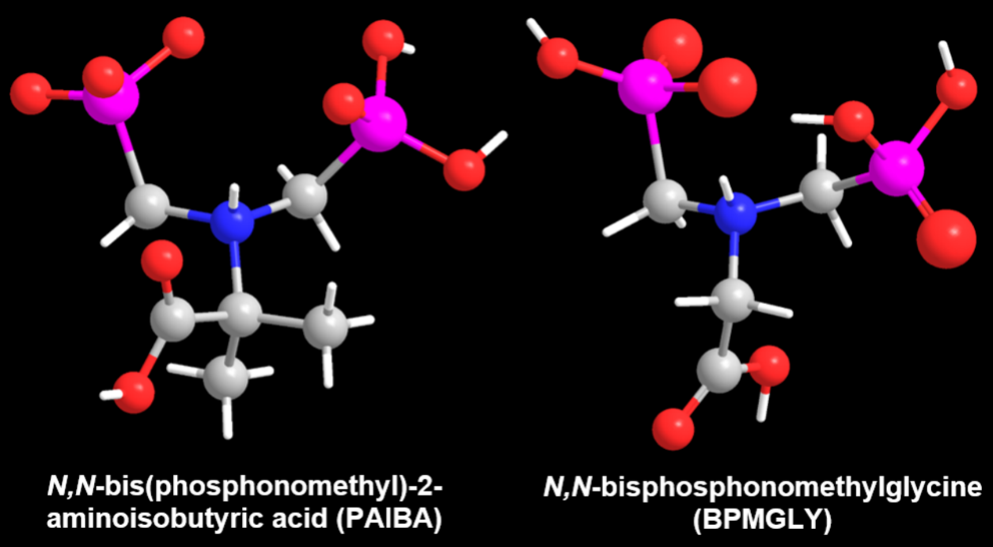
Schematic structures of PAIBA (left) and BPMGLY (right) of their zwitterionic forms. Color codes: O red, C gray, P magenta, N blue, H white.

In this paper we explore the coordination chemistry of the bifunctional tripodal carboxy-diphosphonate PAIBA linker (*N,N*-*bis*(phosphonomethyl)-2-aminoisobutyric acid, Figure 1) with alkaline-earth metal ions that leads to the formation of 0D (Mg), 1D (Sr, Sr/Na), and 3D (Ca) coordination networks, as revealed by single-crystal X-ray crystallography. The latter have been evaluated for their potential as spontaneously formed hybrid coatings for the protection of steel surfaces against corrosion through several techniques (the “standard” gravimetric and electrochemical methodologies). The selection of the alkaline-earth metal ions is based on the fact that they are commonly found in process waters at variable levels, depending on the application. Alongside PAIBA, its analogue BPMGLY (*N,N*-*bis*(phosphonomethyl)glycine, Figure 1), lacking the two −CH_3_ substituents on the β-carbon of the carboxy “arm”, was also explored for its anticorrosion performance. The coordination and structural chemistry of metal-BPMGLY coordination polymers (with the metal ions Ca, Sr, Ba, and Pb) was previously published by our group.^14^ Apart from mapping the unexplored coordination chemistry of PAIBA as a structural analogue of BPMGLY, it would be intriguing to draw structural parallels with the structures presented herein, as well as investigate whether potential structural differences may impact the effectiveness of the anticorrosion coatings.

## Experimental Section

### Materials and Methods

Laboratory-deionized (DI) water from an ion exchange column was used for all of the syntheses. Phosphorous acid (Alfa Aesar, 98% w/w), formaldehyde (37% w/w aqueous solution, stabilized with approximately 10% methanol, Scharlau S.L.), 2-aminoisobutyric acid (Alfa Aesar, 99% w/w), and organic solvents (acetone and ethanol) were purchased from various common commercial sources and were used with no further purification. The starting metal salts (MgCl_2_·6H_2_O, CaCl_2_·2H_2_O, SrCl_2_·6H_2_O, BaCl_2_·2H_2_O) were also from commercial sources and were used as received. Stock solutions of HCl (0.1 and 1.0 M) and NaOH (0.1 and 1.0 M) were used for the pH adjustments. In addition, stock solutions of KOH (0.1 and 1.0 M) were used for the pH adjustment during the synthesis of the compound Sr(PAIBA)(H_2_O)·4H_2_O. The pH meter was a wTw pH315i setup, equipped with a SeTix 41 electrode. All products reported herein were air- and moisture-stable. Yields ranged from 31 to 87% (based on the metal salt).

### Syntheses of the Carboxy-Diphosphonic Acid Corrosion Inhibitors

#### Synthesis of *N*,*N*-*Bis*(phosphonomethyl)-2-aminoisobutyric Acid Monohydrate (PAIBA)

The synthesis of the ligand was previously reported in the literature.^15^ Due to repeated unsuccessful attempts to synthesize the PAIBA ligand according to the published procedure, we followed a different synthetic pathway based on the Moedritzer–Irani reaction. Hence, in a 250 mL two-neck round-bottom flask equipped with a dropping funnel and a condenser, a mixture of 2-aminoisobutyric acid (5.156 g, 0.05 mol) and phosphorous acid (8.200 g, 0.10 mol) was dissolved into a mixture of 5 mL of DI water and 5 mL of concentrated HCl (36.5–38.0% w/v). The mixture was refluxed at 120 °C for 1 h. Subsequently, an aqueous solution of formaldehyde (14 mL, 0.20 mol) was added dropwise to the reaction mixture. Reflux was maintained, and the final product precipitated inside the reaction mixture within ∼3 h. The solid was recovered by filtration, washed with several portions of ethanol, and left in 100 mL of ethanol with vigorous stirring for 1 h. Further purification of the final product was achieved by recrystallization from water, which was finally dried in an oven at 70 °C. Yield: 11.53 g (∼75%). ^1^H, ^13^C, and ^31^P NMR spectra can be found in Figures S1, S2, and S3 in the Supporting Information (SI). The above synthetic procedure is fully reproducible, giving similar yields each time.

#### Synthesis of *N*,*N*-*Bis*(phosphonomethyl)glycine (BPMGLY)

The ligand was prepared according to a well-established Mannich-type phosphonomethylation process, starting with glycine and following published procedures.^16,17^^1^H, ^13^C, and ^31^P NMR spectra can be found in Figures S4, S5, and S6 in the SI. Yields were >80%.

### Synthesis of Alkaline-Earth Metal-PAIBA Compounds

All compounds were obtained by reactions under either hydrothermal or ambient conditions in acidic aqueous solutions (pH range 2.0–3.0). The synthetic procedures given below for the synthesis of metal-PAIBA compounds are fully reproducible. The reported yields are averages of several synthetic attempts. Hybrid materials of the structural analogue BPMGLY with alkaline-earth metal ions were previously reported in the literature by our research group.^14,18^

In this section, we report the syntheses of the following metal phosphonates:
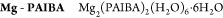

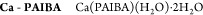

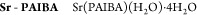

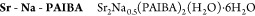


The use of BaCl_2_ under hydrothermal conditions (160 °C for 3 days) in acidic pH values (2.0–4.5) to synthesize a Ba^2+^ analogue led to the hydrolysis of the carboxylate portion of the PAIBA ligand to give amino-*di*(methylenephosphonic acid) [HN(CH_2_PO_3_H_2_)_2_] and 2,2-dimethylacetic acid [(H_3_C)_2_CHCOOH]. The only isolable Ba-containing product was the already published 3D coordination polymer [Ba_3_(O_3_PCH_2_NH_2_CH_2_PO_3_)_2_(H_2_O)_4_]·3H_2_O.^19^ Synthetic efforts at ambient temperature did not give either pure or crystalline products. The syntheses of Mg-, Ca-, Sr-, and Sr-Na- compounds derived from PAIBA are given below, and their attenuated total reflectance infrared (ATR-IR) (Figure S7), powder X-ray diffraction (XRD) (Figure S8), and thermo-gravimetric analysis (TGA) (Figure S9) data are given in the SI.

#### Mg-PAIBA

The compound was synthesized under ambient conditions as follows. A quantity of PAIBA (0.093 g, 0.300 mmol) was placed in 10 mL of DI water, and NaOH stock solution was added dropwise until its complete dissolution (pH = 2). Then, solid MgCl_2_·6H_2_O (0.061 g, 0.300 mmol) was added in portions to the PAIBA solution under stirring. Finally, the pH value of the solution was adjusted to 2.5. The final mixture was covered with parafilm and poked with several holes, and it was left undisturbed for about 10 days at ambient temperature. Colorless crystals were formed due to slow solvent evaporation. They were isolated by filtration, washed with small amounts of DI water, and air-dried. Yield: ∼60% based on the metal salt. Elemental analysis: calculated C 17.082%, H 5.931%, N 3.321%; found C 16.880%, H 5.977%, N 3.299%.

#### Ca-PAIBA

Attempts to synthesize a monophasic crystalline product under ambient conditions failed. Hence, the compound was synthesized under hydrothermal conditions as follows. A mixture of PAIBA (0.015 g, 0.050 mmol) and CaCl_2_·2H_2_O (0.007 g, 0.050 mmol) were dissolved in 3 mL of DI water. The pH was adjusted to 2.8 with HCl and NaOH stock solutions. The clear, colorless solution was placed in a 5 mL Teflon-lined autoclave and heated at 140 °C for 3 days, after which it was cooled to room temperature within about 10 h. The colorless crystalline product was isolated by filtration, washed with small amounts of DI water, and air-dried. Yield: ∼76% based on the metal salt. Elemental analysis: calculated C 18.787%, H 4.958%, N 3.653%; found C 18.692%, H 4.544%, N 3.812%.

#### Sr-PAIBA

The compound was synthesized in a similar manner as Mg-PAIBA (see above), but the adjustment of the pH was performed by KOH (0.1 and 1.0 M stock solutions). Specifically, PAIBA (0.093 g, 0.300 mmol) and SrCl_2_·6H_2_O (0.080 g, 0.300 mmol) were used, and the final pH was adjusted to 2.9. Colorless crystals were formed due to slow solvent evaporation after a period of about 2 weeks. They were isolated by filtration, washed with small amounts of DI water, and air-dried. Yield: ∼31% based on the metal salt. Elemental analysis: calculated C 15.424%, H 4.927%, N 3.000%; found C 16.571%, H 4.719%, N 3.222%.

#### Sr-Na-PAIBA

The compound was initially synthesized in the same manner as Mg-PAIBA (see above). Specifically, PAIBA (0.124 g, 0.400 mmol) and SrCl_2_·6H_2_O (0.106 g, 0.400 mmol) were dissolved in 10 mL of DI water, and the final pH was adjusted to 3.0 with the NaOH stock solution. In this case, the isolated solid contained the main product together with a second phase, which is Sr-PAIBA, Sr(PAIBA)(H_2_O)·4H_2_O. In our attempt to isolate the Sr-Na-PAIBA compound in pure form, a different synthetic pathway was followed. PAIBA (0.045 g, 0.150 mmol) and SrCl_2_·6H_2_O (0.040 g, 0.150 mmol) were dissolved in 5 mL of DI water, and NaOH stock solution was used to adjust the pH value of the final solution to 3.4 with the NaOH stock solution. Subsequently, the solution was transferred to a test tube (14 mm inner diameter), and 5 mL of ethanol was carefully layered on top of the aqueous layer. Over the period of 4 days, the two solvents were fully mixed and pure crystalline Sr-Na-PAIBA was formed, isolated by filtration, washed with small amounts of DI water, and air-dried. Even though the isolated crystalline material was not in a single crystal form, the powder XRD indicates the absence of any secondary phases. Yield: ∼87% based on the metal salt. Elemental analysis: calculated C 16.179%, H 4.382%, N 3.146%; found C 16.126%, H 4.741%, N 3.117%.

### X-ray Crystallography

X-ray diffraction data were collected at room temperature from a single-crystal mounted atop a glass fiber under Paratone-N oil with a Bruker SMART APEX II diffractometer using graphite-monochromated Mo Kα (λ = 0.71073 Å) radiation. The data were processed with the APEX3 suite.^20^ The structures were solved by intrinsic phasing using the ShelXT program,^21^ which revealed the position of all non-hydrogen atoms. These atoms were refined on F^2^ by a full-matrix least-squares procedure using the anisotropic displacement parameter.^22^ Hydrogen atoms were placed at calculated positions and refined using a riding model, except for the water, phosphonic acid, and NH^+^ hydrogens, which were located from the Fourier difference density maps and refined using a riding model with O–H and N–H distance restraints. The Olex2 software was used as a graphical interface.^23^ Molecular graphics were generated using the software Mercury CSD 2.0.^24^ Crystallographic details are summarized in Table S1 in the SI. The structures have been deposited with the CCDC, with the following code numbers: Mg-PAIBA (2314234), Ca-PAIBA (2314235), Sr-PAIBA (2314236), and Sr-Na-PAIBA (2314237).

### Topological Analysis

For further understanding of the metal–organic or H-bonded networks in the obtained compounds, their topological analysis was performed^25,26^ by applying a concept of the simplified underlying net.^25−28^ Simplified nets were constructed by reducing all of the bridging ligands (for analysis of metal–organic networks in Ca-PAIBA, Sr-PAIBA, and Sr-Na-PAIBA) or molecular metal-complex units (for analysis of the H-bonded network in Mg-PAIBA) to the respective centroids while preserving their connectivity. For H-bonded nets, only strong hydrogen bonds were considered with the following parameters: D–H···A: D···A < 3.50 Å, H···A < 2.50 Å, ∠(D–H···A) > 120°; D/A refer to donor/acceptor atoms.^25,26^

### Protocol for the Preparation of Carbon Steel Specimens for Corrosion Studies (Gravimetric Method)

Carbon steel specimens (carbon steel alloy C1010) were used for this study because this metallurgy is widely used in industry. An established experimental methodology was used to calculate corrosion rates based on a reliable protocol issued by the National Association of Corrosion Engineers (USA).^29^ This protocol is being used in our group for the evaluation of corrosion and corrosion inhibition processes.^30^ The synthesized carboxy-diphosphonic acids (BPMGLY and PAIBA) in the absence or presence of metal ions were evaluated for their ability to inhibit metallic corrosion. Hence, a preweighed carbon steel specimen was completely immersed in an aqueous solution containing the inhibitor at preselected concentrations (0.1, 0.5, and 1.0 mM) and at various pH values (4.0, 5.0, and 6.0) for 7 days. The specimens were then removed, mechanically cleaned, and finally weighed in order to calculate the mass loss and hence the corrosion rate. The same conditions were used for experiments in the presence of the carboxy-diphosphonic acid and an alkaline-earth metal ion (Mg^2+^, Ca^2+^, and Sr^2+^, at a metal:phosphonate 1:1 molar ratio) to investigate their synergistic behavior. Appropriate controls (in the absence of inhibitors) were also carried out.

The Corrosion Rate (CR) can be calculated based on eq 1:
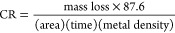
1where CR is expressed in milligrams per year, the mass loss in mg, the area of the metal specimen exposed to the corrosive solution in cm^2^, and the time in hours. The metal density for C1010 is 7.87 g/cm^3^.

The “% inhibition” can be calculated using eq 2:
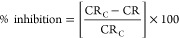
2where CR_C_ is the corrosion rate for the control solution (no additives present), and CR is the corrosion rate for the studied system in the presence of an inhibitor.

### Electrochemical Measurements

Potentiodynamic polarization studies were performed on carbon steel in mildly acidic aqueous solutions (pH ∼ 5) with and without phosphonate inhibitors. The corrosion parameters such as corrosion potential (*E*_corr_), corrosion current (*J*_corr_), polarization resistance (*R*_p_), and corrosion rate (*R*_corr_) were determined. The inhibitor solutions contained each of the phosphonic acids alone (at 5 mM concentration) or a mixture of each with SrCl_2_ at a molar ratio of 1:1 at pH ∼ 5. Triplicate runs were carried out. Electrochemical impedance spectroscopy (EIS) experiments were performed with an Autolab 302N potentiostat/galvanostat equipped with the FRA2 impedance module. All electrochemical measurements were performed at ambient temperature in a one-compartment three-electrode cell equipped with two inox counter electrodes, an Ag/AgCl reference electrode, and a carbon steel working electrode with an exposed surface area of 0.785 cm^2^. EIS spectra were recorded at open-circuit potentials (OCP) in solutions with and without inhibitors. The tested frequency range was from 0.01 Hz to 100 kHz, and the sinusoidal potential amplitude was 10 mV. The experimental data were fitted to the equivalent electrical circuit by a complex nonlinear least-squares procedure using the ZView software by Scribner Associates Inc.

### Vibrational and NMR Spectroscopy and TGA analysis

Attenuated total reflectance infrared (ATR-IR) spectra were recorded with an FT/IR-4200 JASCO spectrophotometer equipped with PIKe ATR (MIRacle), DTGS detector, and Ge crystal plate. These experiments were set at a resolution of 4 cm^–1^ in the range of 4000–600 cm^–1^. All data were analyzed by Spectral Manager Version 2 software. ^1^H, ^31^P, and ^13^C NMR spectra were recorded on a Bruker DPX-300 spectrometer in D_2_O. The solvent residual peak was used as a standard for ^1^H NMR measurements in D_2_O (4.79 ppm), and in ^13^C NMR measurements, CD_3_OD was added as a reference (49.00 ppm). H_3_PO_4_ (85% aqueous solution) was used as an external standard in the ^31^P NMR measurements. Thermogravimetric analysis (TGA) data were recorded on an SDT-Q600 analyzer from TA Instruments. The temperature varied from room temperature (RT) to 900 °C at a heating rate of 10 °C·min^–1^ under an air or N_2_ flow.

## Results and Discussion

### Syntheses of M-PAIBA (M = Mg, Ca, Sr, or Sr/Na) Coordination Networks

The carboxy-diphosphonate PAIBA linker (and the related BPMGLY) exists as a zwitterion in the absence of an externally added base, as one of the phosphonic acid groups (which is more acidic than the carboxylic) internally protonates the basic N atom. They can potentially acquire a maximum “5–” charge, with both phosphonic acid and the carboxylic acid groups completely deprotonated.^15,31^ This can only be achieved at pH > 10, where the N is non-protonated. However, at the synthesis pH values (around ∼ 3), PAIBA carries a “2–” charge: the carboxylic acid group is deprotonated (COO^–^), the two phosphonic acid groups are singly deprotonated (PO_3_H^–^), and the N atom is protonated (NH^+^).

Mildly acidic pH values (<5) have been suitable for obtaining neutral frameworks of aminomethylene-type phosphonate ligands with divalent metal ions. Hence, the following balanced reaction equations can be envisioned for the synthesis of the obtained materials Mg-PAIBA, Ca-PAIBA, Sr-PAIBA, and Sr-Na-PAIBA (the individual protonation states are clearly shown):
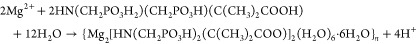

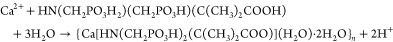

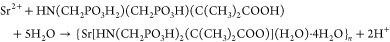

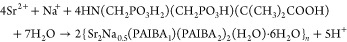

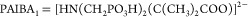

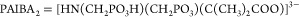


### Description of the Structures

#### Mg-PAIBA

**Figure 2:**
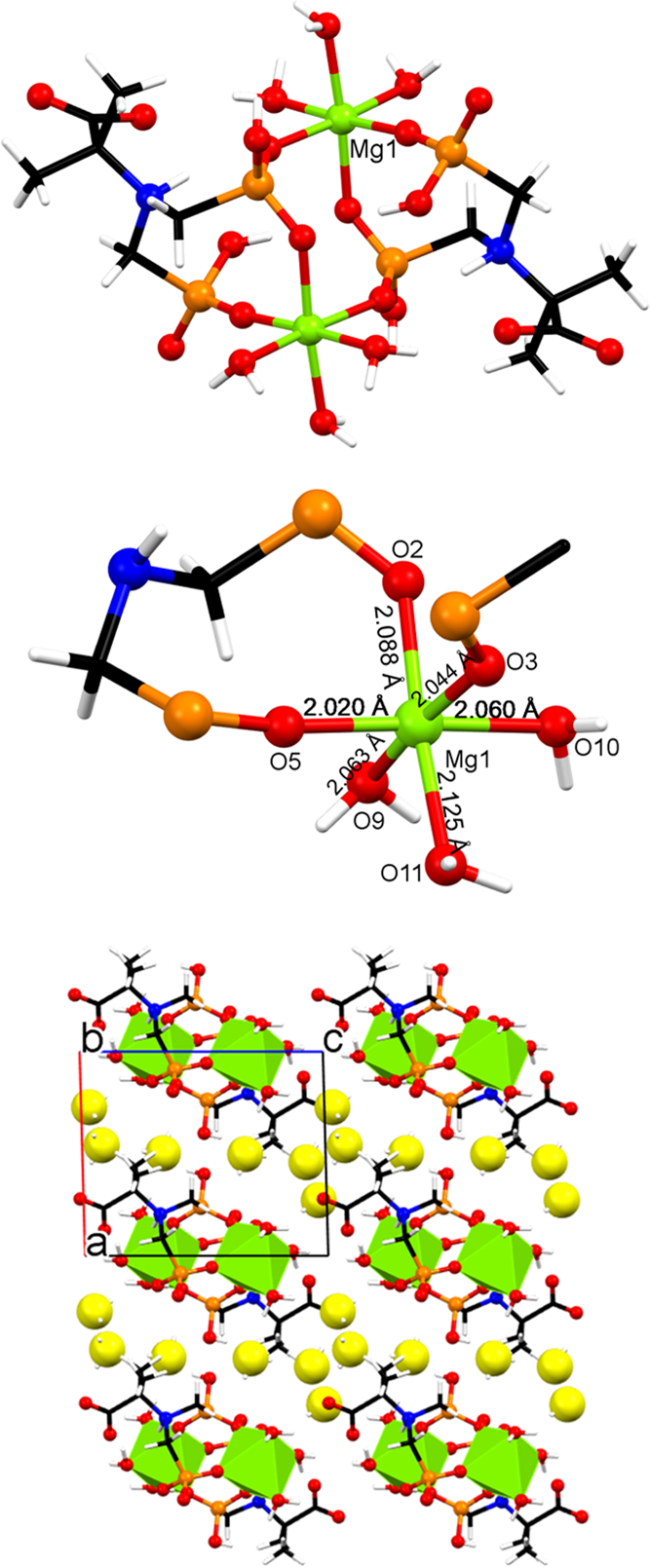
Structural features of the Mg-PAIBA dinuclear complex. (Upper) Basic dimeric unit. (Middle) The coordination environment of the octahedral Mg center with M–O bond distances. (Lower) Packing of the dinuclear units along the *b*-axis. The lattice water molecules are displayed as exaggerated yellow spheres. Color codes: Mg, green; P, orange; C, black; O, red; and H, white.

This is a doubly bridged neutral dinuclear complex; see Figure 2. Both Mg centers are crystallographically equivalent and are located in a nearly perfect octahedral environment according to SHAPE (Figure 2, upper).^32^ Each Mg center is coordinated by three phosphonate oxygen atoms (O2, O3, and O5) and three water ligands (O9, O10, and O11). Two of the phosphonate O’s (O2, O5) originate from the same PAIBA ligand and form an 8-membered chelate ring. The third (O3) comes from a neighboring PAIBA ligand and is terminal. The O2-P1-O3 moiety acts as a bridge between the two Mg centers. The three Mg-coordinated water ligands are in a *fac* configuration. The N atom of each PAIBA ligand is protonated, while each phosphonate group is monodeprotonated. This causes each PAIBA ligand to carry a total “2–” charge, and thus, electroneutrality is achieved. The carboxylate moiety is deprotonated (−COO^–^) and noncoordinating. The six lattice water molecules are found between the dinuclear units and serve to create a complex network of H-bonds. The Mg–O bond distances are found in the range of 2.020–2.125 Å (Figure 2, middle) and are within the expected range found in other Mg-phosphonate compounds.^18^ The dimeric units interact through H-bonds, and the Mg···Mg separation is 9.505 Å (parallel to the *a*-axis). The six lattice water molecules are found between the dinuclear units (Figure 2, lower) and further contribute to the formation of a complex 3D network of H-bonds with the 6,10T9 topology (*vide infra*).

#### Ca-PAIBA

**Figure 3:**
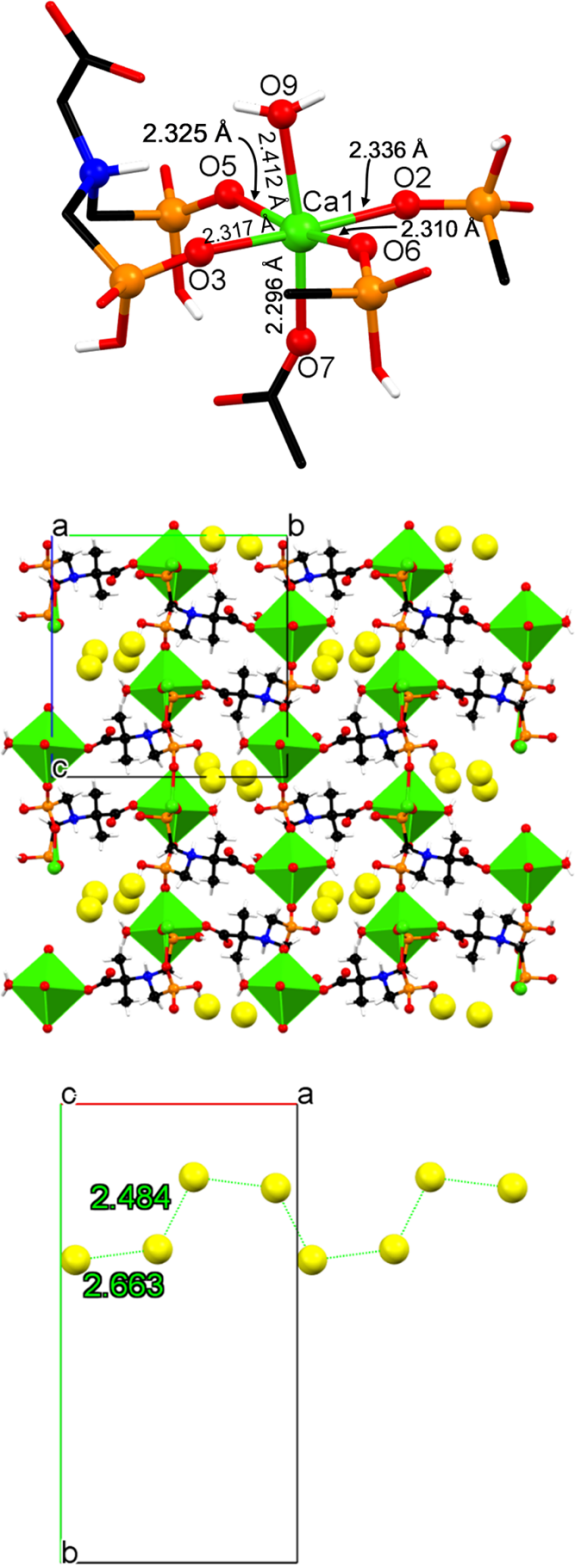
Structural features of 3D coordination polymer Ca-PAIBA. (Upper) The coordination environment of the octahedral Ca center with Ca–O bond distances. (Middle) Packing of the structure along the *a-*axis. The lattice water molecules are displayed as exaggerated yellow spheres. (Lower) The zigzag chain of the lattice water molecules is seen along the *a*-axis. Color codes: Ca, green; P, orange; C, black; O, red; and H, white.

This is a 3D coordination polymer; see Figure 3. The Ca ion is found in an octahedral environment formed by exclusively O atoms (Figure 3, upper), according to SHAPE.^32^ Specifically, the equatorial ligands are phosphonate O donors. Two oxygen atoms from the same PAIBA ligand but from different phosphonate groups (O3 and O5) form an 8-membered chelate ring (just like in the Mg-PAIBA case). The other two (O2 and O6) originate from two neighboring PAIBA ligands and are coordinated in a terminal fashion. One of the axial ligands is a monodentate carboxylate (O7), and the other is a water molecule (O9). The Ca–O bond distances are in the range of 2.296–2.412 Å (Figure 3, upper), which is within the expected values found in other Ca-phosphonate compounds.^33^ The N atom of each PAIBA ligand is protonated, while each phosphonate group is monodeprotonated. This causes each PAIBA ligand to carry a total “2–” charge, and thus electroneutrality is achieved. The carboxylate moiety is deprotonated (−COO^–^) and coordinates with the Ca center (in contrast to that in Mg-PAIBA). The structure of Ca-PAIBA could be envisioned as a 2D layered motif, with each layer containing an inorganic part (the Ca octahedra) and an organic part (the PAIBA dianion). However, these layers are further connected via the O2-P1-O3 bridges to create the 3D network (Figure 3, middle) of the **unc** topological type (*vide infra*). Upon this tethering, one-dimensional channels that run along the *a*-axis are created. These are filled with the lattice water molecules that form a chain and interact with each other via H-bonds (O···O distances of 2.484 and 2.663 Å, Figure 3, lower) to provide additional stabilization of 3D metal–organic assembly.

#### Sr-PAIBA

**Figure 4:**
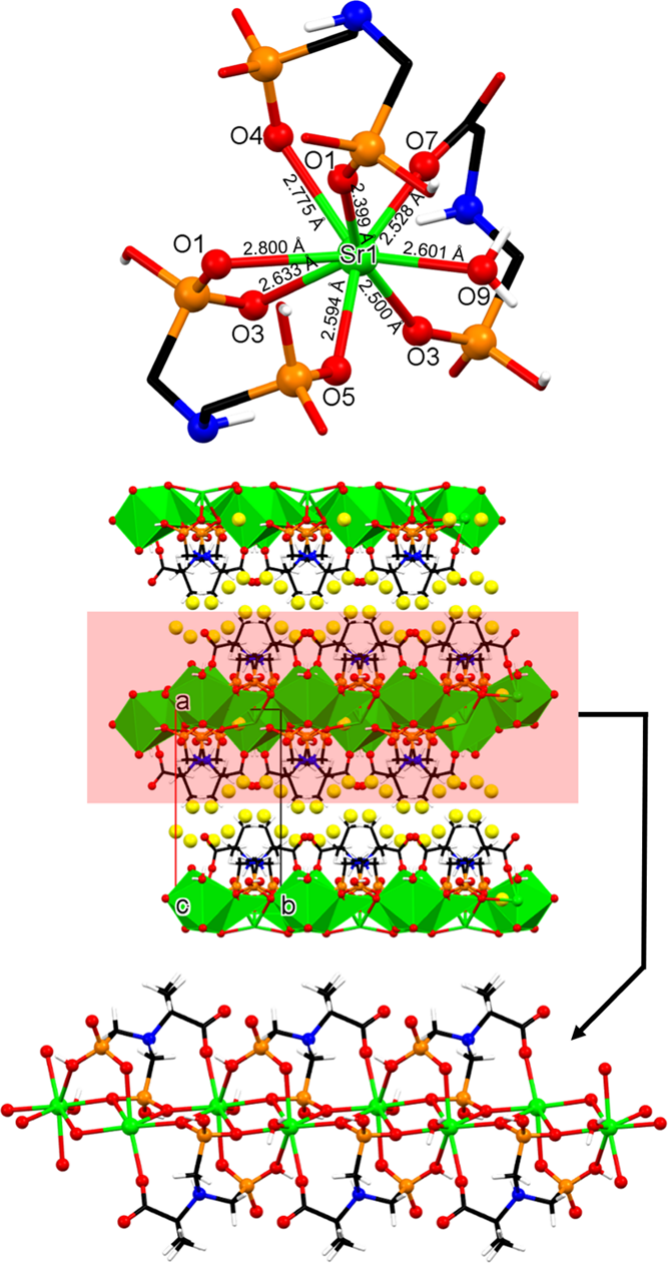
Structural features of the 1D coordination polymer Sr-PAIBA. (Upper) The coordination environment of the 8-coordinated polyhedral center with Sr–O bond distances. (Middle) Packing of the structure along the *c*-axis. The lattice water molecules are displayed as exaggerated yellow spheres. (Lower) Portion of a single 1D chain. Color codes: Sr, green; P, orange; C, black; O, red; and H, white.

This is a 1D coordination polymer (Figure 4). The Sr ion is found in an 8-coordinated environment, formed by exclusively O atoms (Figure 4, upper), defined as a triangular dodecahedron environment based on SHAPE.^32^ One of the ligands is a water molecule (O9). The remaining coordinated atoms originate from three different PAIBA ligands. Specifically, there are two *bis*-chelating and one tris-chelating PAIBA dianions. The O1 and O4 are phosphonate oxygen atoms and come from the same PAIBA ligand but from different phosphonate groups and form an 8-membered chelate ring (just like in the Mg-PAIBA and Ca-PAIBA). The other bis-chelating PAIBA ligand utilizes a phosphonate oxygen (O3) and a carboxylate oxygen (O7). The third, tris-chelating PAIBA offers two oxygen atoms from the same phosphonate group (O1 and O3) and a third oxygen atom (O5) from its second phosphonate group. The Sr–O bond distances show great variability and are in the range of 2.399–2.800 Å (Figure 4, upper), being within the expected values reported for other Sr-phosphonate compounds.^34^ The N atom of each PAIBA ligand is protonated, while each phosphonate group is monodeprotonated. This causes each PAIBA ligand to carry a total “2–” charge, and thus electroneutrality is achieved. The carboxylate moiety is deprotonated (−COO^–^) and coordinates to the Sr center. Adjacent 1D chains in Sr-PAIBA feature a common SP 1-periodic net (4,4)(0,2) topology (*vide infra*) and are extended into a complex H-bonded network via multiple hydrogen bonds involving crystallization water molecules (Figure 4, middle and lower).

#### Sr-Na-PAIBA

**Figure 5:**
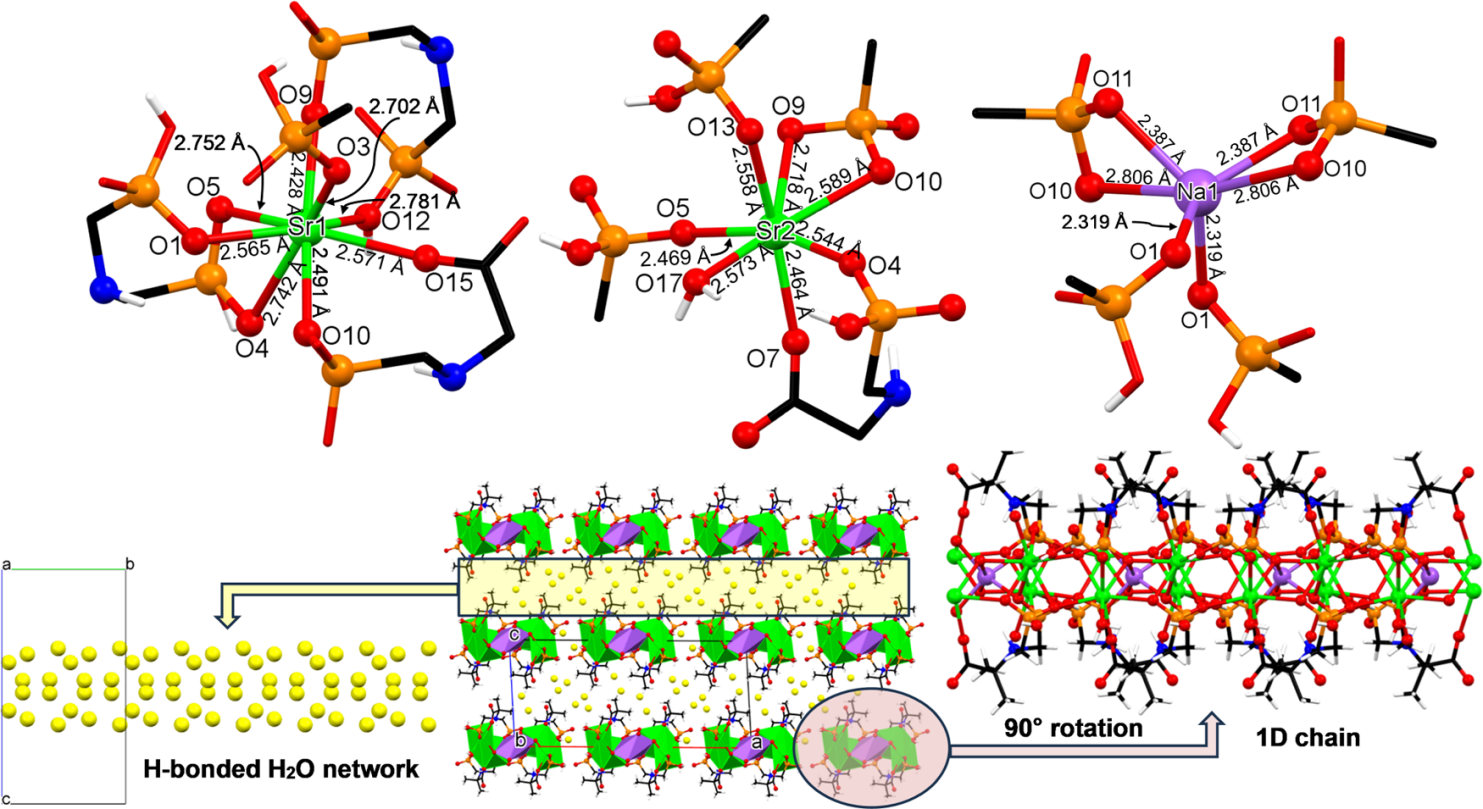
Structural features of the 1D coordination polymer Sr-Na-PAIBA. (Upper) The coordination environments of the two types of Sr polyhedral centers, the 8-coordinated (Sr1, left) and the 7-coordinated (Sr2, middle), and the 6-coordinated Na center (right), with Sr–O and Na–O bond distances. (Lower) Packing of the structure along the *b*-axis (middle). The H-bonded lattice water molecules are displayed as exaggerated yellow spheres (left). A single 1D chain (right). Color codes: Sr, green; P, orange; C, black; O, red; and H, white.

This compound is also a 1D coordination polymer (Figure 5), which, however, features a significantly more complex structure and topology when compared to Sr-PAIBA. This is governed by the presence of two types of crystallographically distinct Sr centers, one 8-coordinated (Sr1, Figure 5, upper left) and one 7-coordinated (Sr2, Figure 5, upper middle), along with an additional Na center.

#### Sr1 Center

The coordination environment of Sr1 is a triangular dodecahedron, according to SHAPE.^32^ There are two bis-chelating and one tris-chelating PAIBA dianions and a terminal phosphonate oxygen (O3) (Figure 5, upper left). O9 and O12 are phosphonate oxygen atoms and come from the same PAIBA ligand but from different phosphonate groups and form an 8-membered chelate ring (just like in the Mg-, Ca-, and Sr-PAIBA cases). The other bis-chelating PAIBA ligand utilizes a phosphonate oxygen (O10) and a carboxylate oxygen (O15). The third, tris-chelating PAIBA offers two oxygen donors from the same phosphonate group (O4 and O5) and a third oxygen (O1) from its second phosphonate group. The Sr–O bond distances show great variability and are in the 2.428–2.781 Å range.

#### Sr2 Center

The coordination environment of Sr2 is a capped trigonal prism, according to SHAPE.^32^ There are two bis-chelating groups, two terminal phosphonates (O5 and O13), and a water molecule (O17) in the coordination sphere of Sr2 (Figure 5, upper middle). The bis-chelating groups are very different based on the size of the formed ring. The O4 and O7 are phosphonate and carboxylate oxygen donors, respectively, and come from the same PAIBA ligand, forming an 8-membered chelate ring (just like in the Mg-, Ca-, and Sr-PAIBA cases). The other bis-chelating group is from the same phosphonate group (O9 and O10) and forms a 4-membered ring. The Sr–O bond distances show great variability and are found in the range of 2.464–2.718 Å.

#### Na Center

The 6-coordinated geometry of the Na center is peculiar (Figure 5, upper left) and could be described as a trigonal prismatic environment, according to SHAPE.^32^ Apparently, this distortion is related to the presence of the two identical 4-membered rings (O10 and O11 oxygen atoms in both). The coordination environment of Na is completed by two terminal phosphonate oxygens (O1). Na–O bond distances vary dramatically and are in the range of 2.319–2.806 Å. As in the previous structures, the formation of an extensive intermolecular hydrogen-bonding network gives rise to the extension of the 1D chains into a 3D hydrogen-bonded net.

### Topological Analysis of the Metal-PAIBA Compounds

#### Mg-PAIBA

**Figure 6:**
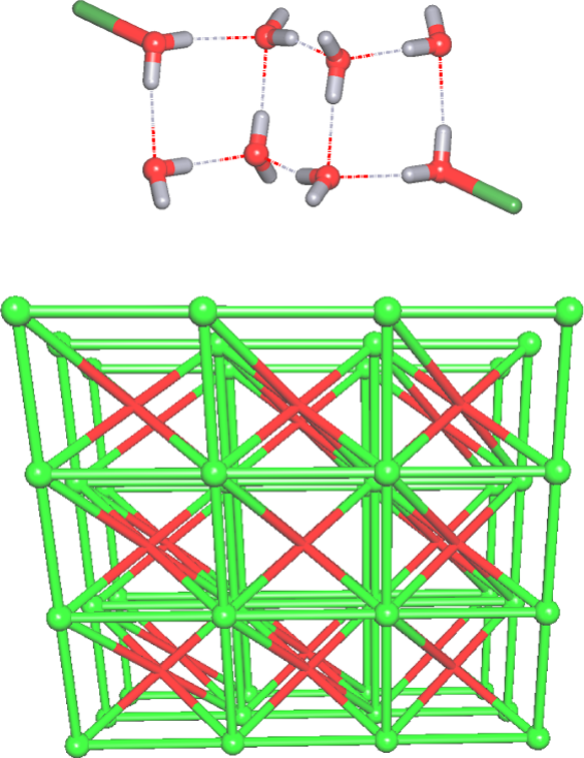
(Upper) Water cluster in Mg-PAIBA. (Lower) Topological representation of a binodal 6,10-connected 3D H-bonded framework with the 6,10T9 topology in Mg-PAIBA; perspective view along the *a-*axis; centroids of 10-connected Mg_2_ molecular units (green spheres), centroids of 6-connected (H_2_O)_6_ clusters (red).

The discrete Mg_2_ molecular units and six crystallization water molecules are multiply H-bonded into a very complex 3D H-bonded network. Within this network, the (H_2_O)_6_ clusters with a cyclic (H_2_O)_4_ core and two dangling water molecules can be identified. If terminal water ligands are taken into consideration, an overall tricyclic (H_2_O)_8_ aggregate is formed (Figure 6, upper). By treating the (H_2_O)_6_ clusters as the 6-connected nodes and the Mg_2_ molecular units as the 10-connected molecular nodes, a binodal 6,10-connected framework can be generated (Figure 6, lower). This 3D H-bonded framework features the 6,10T9 topology and the point symbol of (3^4^.4^8^.5^3^)(3^8^.4^20^.5^12^.6^5^).

#### Ca-PAIBA

**Figure 7:**
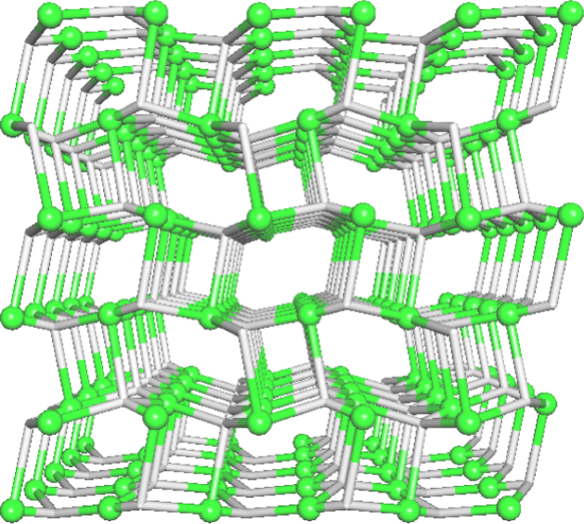
Topological representation of a uninodal 4-connected 3D net with the **unc** topology in Ca-PAIBA; perspective view along the *a*-axis; 4-connected Ca1 nodes (green spheres), centroids of 4-connected μ_4_-PAIBA spacers (gray).

This 3D coordination polymer structure is driven by the topologically equivalent 4-connected Ca1 and μ_4_-PAIBA nodes (Figure 7). The resulting net can be described as a uninodal 4-linked framework with the **unc** topology and point symbol of (6^6^).

#### Compounds Sr-PAIBA and Sr-Na-PAIBA

**Figure 8:**
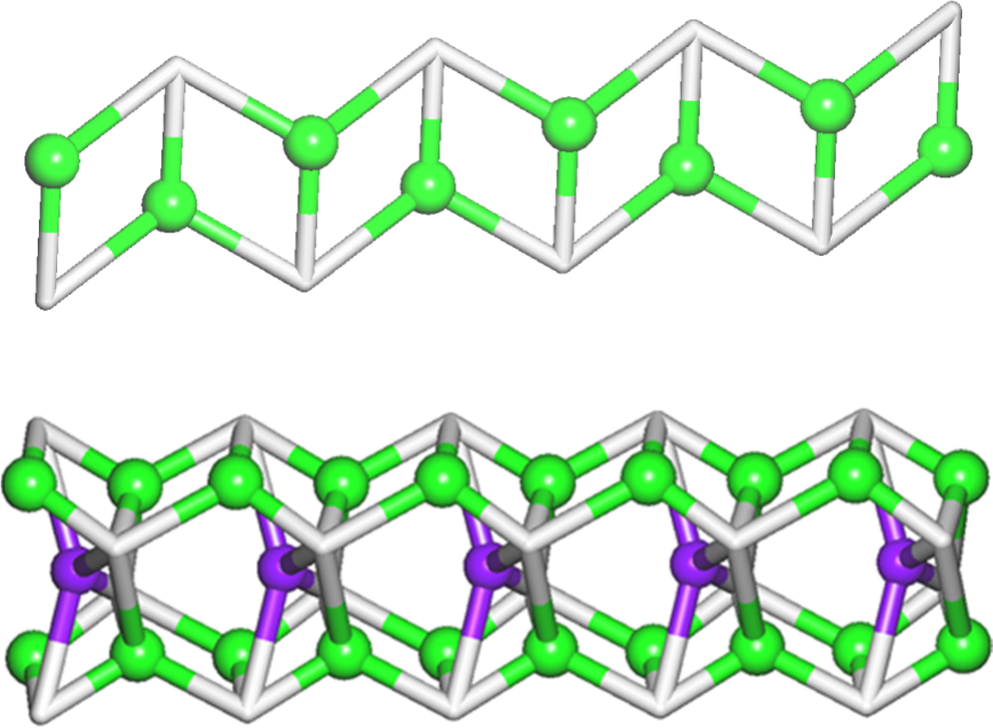
Topological representations of: (Upper) A uninodal 3-connected 1D net in Sr-PAIBA with an SP 1-periodic net (4,4)(0,2) topology; (Lower) A tetranodal 3,4,5-connected 1D net in Sr-Na-PAIBA with a unique topology. Both images are rotated views along the *c*-axis. Sr nodes (green spheres), Na nodes (purple spheres), and centroids of μ_3–5_-PAIBA spacers (gray).

Topological classification of the 1D metal–organic chain in Sr-PAIBA reveals a uninodal three-linked net composed of topologically equivalent Sr1 and μ_3_-PAIBA nodes (Figure 8, upper). This chain features an SP 1-periodic net (4,4)(0,2) topology and the point symbol of (4^2^.6).

From a topological perspective, the 1D coordination polymer Sr-Na-PAIBA is significantly more complex (Figure 8, lower) and composed of the three-linked Sr2 and four-linked Sr1 and Na1 nodes, as well as the 4- and five-connected μ_4_- and μ_5_-PAIBA nodes, thus giving rise to a very intricate tetranodal 3,4,5-connected net with a unique topology. It is described by the point symbol of (4^2^.6)_2_(4^4^.6^2^)_4_(4^5^.6)(4^6^.6^4^)_2_, wherein the (4^2^.6), (4^4^.6^2^), (4^5^.6), and (4^6^.6^4^) notations correspond to the Sr2, Sr1/μ_4_-PAIBA (topologically equivalent), Na1, and μ_5_-PAIBA nodes, respectively.

Summarizing the topological part, it should be mentioned that the variety of topological types identified in the studied crystal structures is primarily explained by the differences in coordination modes of PAIBA ligands and overall network dimensionality, as well as by the presence of several crystallographically distinct metal centers. The latter contributes to a topological complexity, leading to even previously undisclosed topologies, as in the case of Sr-Na-PAIBA. Although no obvious correlation between structural and topological type and the anticorrosion performance can be drawn, it is expected that the materials possessing less stabilized networks and featuring lower dimensionality (e.g., Sr-PAIBA) may exhibit some advantages.

### Further Physicochemical Characterization of the Metal-PAIBA Compounds

#### Thermogravimetric Analysis (TGA)

All TGA traces are given in Figure S9 in the SI. The dinuclear unit in Mg-PAIBA [Mg_2_(PAIBA)_2_(H_2_O)_6_·6H_2_O] contains six lattice and six Mg-bound water molecules (three on each Mg). A mass loss of 4.40% is noted up to a temperature of ∼50 °C, which corresponds to the removal of two, most likely, lattice water molecules (calculated 4.27%). Upon temperature increase to up to 150 °C an additional 18.91% loss is observed, which corresponds to the removal of nine water molecules (calculated 19.21%). The last water molecule is removed from the dimer upon further heating but overlaps with ligand decomposition. The coordination polymer Ca-PAIBA [Ca(PAIBA)(H_2_O)·2H_2_O] contains two lattice and one Ca-bound water molecules. Heating up to ∼90 °C causes the loss of one lattice water molecule (calculated 4.70%, measured 5.52%). The second lattice water molecule is removed at ∼198 °C (calculated 4.70%, measured 5.72%). These two lattice water molecules are removed at different temperatures due to the different number of hydrogen-bonding interactions they develop in the lattice. One water molecule (O10) interacts via four, whereas the other (O11) via three hydrogen bonds with neighboring atoms. Finally, the Ca-bound water is removed at higher temperatures during a step that coincides with ligand decomposition. The molecular formula of Sr-PAIBA [Sr(PAIBA)(H_2_O)·4H_2_O] contains four lattice and one Sr-coordinated water molecules (19.29 wt %). These are removed in two ill-defined steps (up to ∼260 °C, ∼20% mass loss). Temperature increase leads to ligand decomposition. Based on the molecular formula of Sr-Na-PAIBA [Sr_2_Na_0.5_(PAIBA)_2_(H_2_O)·6H_2_O], there are six lattice and one Sr-coordinated water molecules (14.17 wt %). A weight loss of 13.99% is noted in the TGA trace up to ∼150 °C, which corresponds to the loss of all seven water molecules in one step. Temperature increase leads to further weight loss, most likely due to ligand decomposition.

#### Powder XRD

Comparative powder X-ray diffraction diagrams (calculated vs measured) of all M^2+^-PAIBA compounds are given in Figure S8 in the SI. The experimentally measured diffractograms are in satisfactory agreement with the calculated ones.

#### Vibrational Spectroscopy

All ATR-IR spectra are given in Figure S7 in the SI. The PAIBA linker contains two types of functional moieties: one carboxylate and two phosphonates. Upon metal coordination, various changes and shifts occur in the peaks associated with these groups. The most profound shift is observed for the ν(C=O) asymmetric stretch, which appears at 1717 cm^–1^ in “free” PAIBA, as expected for a protonated and uncoordinated −COOH group. Upon metal coordination, this band shifts to lower frequencies. In Mg-PAIBA, it appears at 1602 cm^–1^, in Ca-PAIBA at 1605 cm^–1^, in Sr-PAIBA at 1614 cm^–1^, and in Sr-Na-PAIBA at 1626 cm^–1^. In all compounds, the carboxylate group is deprotonated and metal coordinated, except in Mg-PAIBA, in which it is deprotonated and unbound. The range 900–1200 cm^–1^ is a complex spectral region characteristic of vibrations related to the −PO_3_ moiety. It is a useful “fingerprint” region but challenging to fully assign. Its practical usefulness is the shifts of various peaks of the ligand upon metal coordination. Peaks at <600 cm^–1^ are assigned to the M–O (phosphonate, carboxylate) bond stretches. All compounds show a weak and broad peak at ∼2300 cm^–1^, which is assigned to the N–H^+^ moiety. The weak peaks noted in the region of 3000–3100 cm^–1^ are assigned to the C–H vibrations of the −CH_2_– and −CH_3_ groups. The O–H vibrations appear in the range 3100–3400 cm^–1^ and originate from the water molecules (metal coordinated and in the lattice).

## Corrosion Inhibition

### Long-Term Performance of M-PAIBA and M-BPMGLY (M = Mg^2+^, Ca^2+^, Sr^2+^, and Ba^2+^) Synergistic Systems Based on Gravimetric Measurements

Each of the PAIBA and BPMGLY was tested in the presence of alkaline-earth metal ions Mg^2+^, Ca^2+^, Sr^2+^, and Ba^2+^ (separate solutions of the phosphonates and the metal ions were mixed together in the same container) at three pH values, *i.e.*, 4.0, 5.0 (same as the electrochemical measurements), and 6.0, and at concentrations 0.1, 0.5, and 1.0 mM (equimolar to the M^2+^). Visual inspection of the “control” specimen (no inhibitors) after air-drying showed general corrosion on the specimen surface (see the “control” images in Figures S10–S15 in the SI). In contrast, the steel revealed a protective coating on the surface when phosphonates were present. Figures S10–S15 in the SI show all specimens for comparison. The measured corrosion rates and calculated inhibitory efficiency (%) are provided in Tables S2 (for the BPMGLY systems) and S3 (for the PAIBA systems) in the SI. Inhibition performance graphs on each individual system are provided in Figures S16–S18 in the SI.

The optical images of the carbon steel surface after immersion (in the absence or presence of inhibitors, Figures S10 and S15) warrant some discussion. The metal surface was substantially covered by corrosion products in the case of the “control” at all pH values. However, the trend in the corrosion rates showed higher values as the pH was lower, as expected. All corrosion inhibitor systems demonstrate variable corrosion inhibition. The influence of inhibitor concentration on anticorrosion performance (at least based on the visual) is difficult to ascertain. This is because at the two lower inhibitor concentrations (0.1 and 0.5 mM), all specimens show the presence of corrosion products, whereas at the highest concentration (1.0 mM), the specimens appear to be free of corrosion products; however, the corrosion rate values are comparable. This can be rationalized as follows. At the two lower inhibitor concentrations (e.g., see specimens for Sr-BMPGLY, Mg-PAIBA, Ca-PAIBA, and others), coating formation is only partial, as evidenced visually and based on % inhibition (30–60%), without any obvious differentiation for the two concentrations. However, a dramatic change is noted when the inhibitor concentration is increased to 1.0 mM. All carbon steel specimens appear “clean” from corrosion products, albeit the corrosion efficiencies do not seem to increase. We propose that, in this case, a secondary competitive reaction takes place, the dissolution of Fe oxy/hydroxides by the phosphonate additives. It seems that at high dosages, the phosphonate (BPMGLY or PAIBA) performs corrosion protection when at the same time it interacts with the oxidized surface by coordination with the exposed Fe sites. Due to the high affinity of phosphonates to ferric ions (in general), Fe-phosphonate “complex” formation can be envisioned.^35^ These complexes can then depart from the surface and become solubilized. This action is expected to increase corrosion rates as the surface is depleted from Fe. The overall result that is reflected in corrosion rates and % inhibition is the combination of the two competing events: surface passivation and surface dissolution. This phenomenon has been observed before in the corrosion protection of carbon steel by the Ca-PBTC inhibitor system (PBTC = 2-phosphonobutane-1,2,4,-tricarboxylic acid).^36^

In addition to these qualitative visual observations, corrosion rates and inhibition efficiencies (%) were calculated based on mass loss measurements. The results are presented in Tables S2 and S3 in the SI. Some discussion is warranted on the influence of four variables, i.e., the nature of the inhibitor (PAIBA vs BPMGLY), the identity of the alkaline-earth metal ion present, the solution pH, and the concentration of the inhibitor. Based on these long-term inhibition results, there appear to be no systematic trends in the performance characteristics of the two carboxy-diphosphonate inhibitors. This may be due to a number of reasons. The long carbon steel specimen exposure (1 week) to the metal phosphonate aqueous solution may allow other competing phenomena to take place, such as Fe oxide dissolution by the additives (particularly at the high concentration 1.0 mM). This would lead to mass loss and increased corrosion rates. Oxide dissolution by the carboxy-diphosphonates would generate Fe-inhibitor “complexes” in solution, which may reprecipitate on the steel surface in an unpredictable manner. Fe-phosphonate adducts are known to have low solubilities.^37^ In this case, an added complication is the unknown solubility of the Fe-diphosphonates formed in solution or reprecipitated on the steel surface. Hence, the mass loss results should be received with caution, taking into account the unpredictability of this system, at least in the long term (1 week duration of the mass loss experiments), which may allow sufficient time for several competing phenomena to take place. Nonsystematic variations in corrosion rates (based on mass loss) have been observed in the tetraphosphonate systems reported by us recently,^38^ but to a lesser extent.

**Figure 9:**
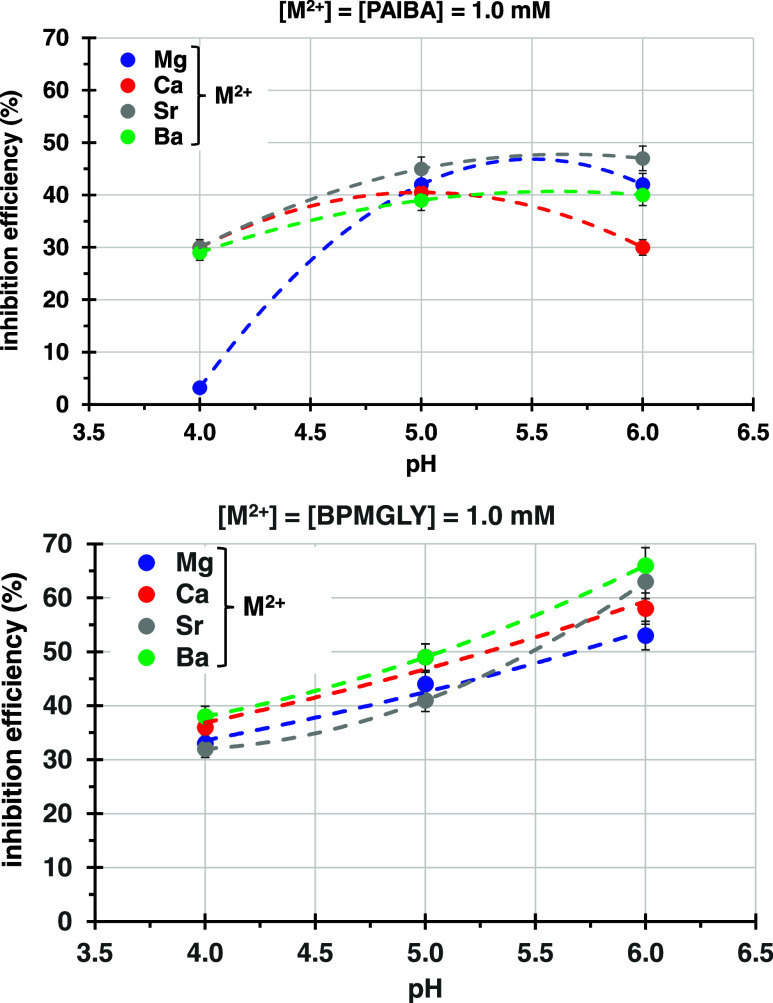
Dependence of inhibition efficiency on the system pH and the metal ion present: M^2+^-PAIBA (upper) and M^2+^-BPMGLY (lower) (M = Mg, Ca, Sr, and Ba). Lines are drawn to aid the reader.

Figure 9 shows a selected example of the effect of pH on anticorrosion efficiency for both PAIBA (Figure 9, upper) and BPMGLY (Figure 9, lower) systems in the presence of metal ions ([M^2+^] = [phosphonate] = 1.0 mM). For the metal-BPMGLY system, it is evident that as the pH increases, the % inhibition systematically improves, roughly 10 percentage units per pH unit. For the metal-PAIBA system, the same increase, albeit milder, is noted for all inhibitor systems up to pH = 5.0 and for the Sr- and Ba-PAIBA systems up to pH = 6.0, whereas no increase is observed for the Mg-PAIBA and a slight decrease for the Ca-PAIBA.

The effect of the nature of the metal ion is more obvious in the metal-BPMGLY system, although the differences are not dramatic. The Ba-BPMGLY inhibitor appears to be the most effective in all three pH values. The available data are not sufficiently persuasive to suggest specific trends. The metal-PAIBA systems seem to be more sensitive to the nature of the metal ion. For example, Mg-PAIBA at pH = 4.0 is the worst inhibitor (<5% efficiency), with all of the other Ca-, Sr-, and Ba-PAIBA systems showing the same performance (∼30%). Upon pH increase to 5.0, all four systems show comparable performance (39–45%). Then, when pH = 6.0, there is some differentiation, with the Ca-PAIBA showing a slight reduction in performance (30%) and the remaining systems showing a minor improvement (40–47%).

To further characterize the surface of the carbon steel specimens, the following experiments were set up at pH 6: Control (no inhibitor present), PAIBA (1 mM), BPMGLY (1 mM), Sr^2+^+PAIBA (1 mM each), and Sr^2+^+BPMGLY (1 mM each). The steel surfaces were exposed to each solution for ∼10 days, and after they were removed from the solution, they were mildly washed and dried in the oven. Their surface (see Figure S19 in the SI) was analyzed by Electron Dispersive Spectrometry to detect and quantify Sr (from externally added Sr^2+^) and P (from PAIBA or BPMGLY) present in the coatings. The use of powder XRD to possibly identify the compounds Sr-PAIBA and Sr-BPMGLY failed. All surfaces showed the presence of Fe, as expected. The control specimen showed the presence of only Fe and O from the iron oxide corrosion products. Only P (and Fe) was detected on the surfaces in the presence of PAIBA and BPMGLY (in the absence of Sr^2+^). When both Sr^2+^ and PAIBA or BPMGLY are present, Sr and P are detected in the coating formed on the surface. In the case of the Sr^2+^/PAIBA, the P/Sr atom ratio was ∼3.9, much higher than the P/Sr ratio of 2:1 for the Sr-PAIBA compound (no Na was detected). The presence of excess P is a strong indication that PAIBA can interact with Sr^2+^ to form the hybrid coating but can also independently interact with the Fe-oxide layer, possibly forming Fe–O–P surface bonds. In the case of the Sr^2+^/BPMGLY, the P/Sr atom ratio was ∼2.1, close to the P/Sr ratio of 2:1 for the Sr-BPMGLY compound.^14^ This is an indication that BPMGLY shows a preference for Sr^2+^ ions and forms stoichiometric Sr-BPMGLY coatings on the surface. The different behavior of PAIBA can be rationalized by considering its higher acidity due to the inductive effect of the two −CH_3_ substituents, allowing it to react with surface Fe–OH groups in an acid–base reaction.

Overall, all BPMGLY-containing inhibitor blends consistently perform better in long-term experiments than the PAIBA-containing ones, but only slightly. Based on the gravimetric results, we selected two systems, Sr^2+^-BPMGLY and Sr^2+^-PAIBA, to evaluate in more detail with electrochemical techniques. These studies and the collected results are discussed in the following section.

### Electrochemical Measurements: Focus on the Sr^2+^/PAIBA and Sr^2+^/BPMGLY Inhibitor Systems

PAIBA or BPMGLY and their blends with Sr^2+^ can function as corrosion inhibitors due to the formation of a protective film capable of reducing metal loss. The corrosion efficiency depends on the ability to form a stable and compact film on a metal surface. The lowest corrosion rates were noted for steel specimens immersed in mixtures of phosphonic acids and Sr^2+^. This suggests a synergism between the phosphonic acid and the metal cation that allows the phosphonate moiety to bind more strongly to the metal with efficient packing on the surface. This is consistent with results reported for other inhibitors.^39−41^

#### OCP and Potentiodynamic Polarization Testing

**Figure 10:**
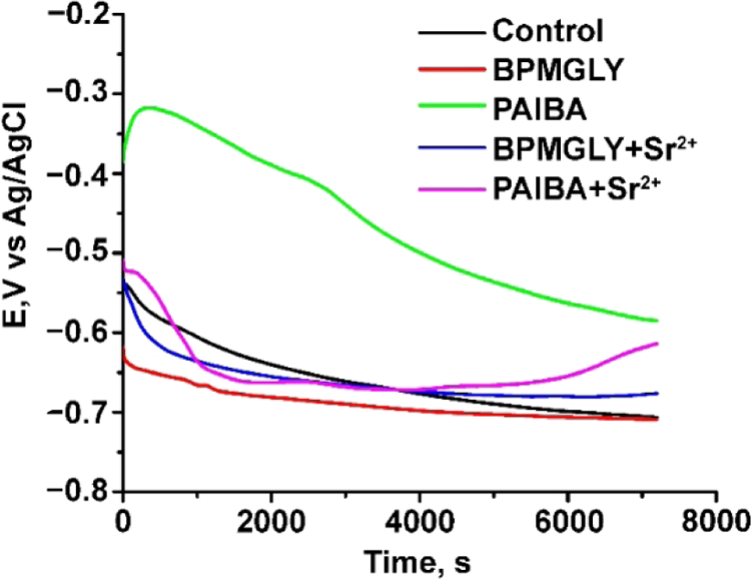
Variation of the OCP with immersion time for carbon steel in acid aqueous solution (pH ∼ 5) in the absence (control) and presence of various inhibitor systems, as indicated.

The time-dependent variation of the OCP of carbon steel immersed in solution with pH ∼ 5 with different inhibitor systems is presented in Figure 10.

It was observed for the metallic surface without inhibitors that the OCP value decreased in time as a result of the dissolution of the passive oxide layer formed at the sample surface (electrodes). In time, partial protection of the surface takes place due to the formation of corrosion products, which are capable of blocking the existing pores and cracks on the surface, leading to a decrease in the corrosion rate and potential stabilization. The OCP of carbon steel immersed in solution without additives reached a constant value after ∼35 000 s. The shorter time needed for OCP stabilization in the presence of inhibitors, compared with that in their absence, suggests the formation of a protecting stratum on the carbon steel. In the presence of BPMGLY, the OCP reached a constant value after 2000 s. The addition of Sr^2+^ in the BPMGLY solution presents the same trend for OCP evolution in time but moves the initial OCP value to higher potential comparatively with carbon steel immersed only in the BPMGLY solution. For carbon steel immersed in an inhibitor system based on PAIBA, the OCP tends to grow during the first ∼350 s, implying the coverage of the metallic surface with inhibitor molecules. The inhibitor molecules are not tightly attached to the metal surface, and after 400 s, the OCP starts to decrease as the inhibitor layer reduces in size. The addition of Sr^2+^ in the PAIBA solution is capable of stabilizing the OCP, and after 4000 s, the layer becomes more compact (the binding to the surface is amplified) and, as a result, the OCP increases.

**Figure 11:**
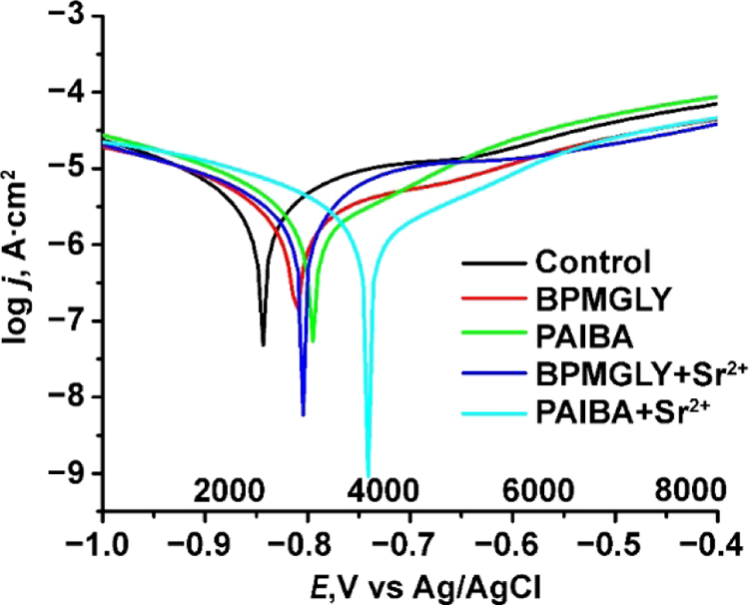
Potentiodynamic polarization curves for carbon steel after 120 min immersion in aqueous solutions (pH ∼ 5) without (control) and with inhibitors present, as indicated (scan rate = 1 mV/s).

The potentiodynamic polarization curves obtained for carbon steel after 120 min immersion in an aqueous solution of pH ∼ 5 with and without the inhibitor systems are shown in Figure 11.

The electrochemical parameters cathodic (β_c_) and anodic (β_a_) Tafel slope, corrosion potential (*E*_corr_), corrosion current density (*J*_corr_), polarization resistance (*R*_p_), and corrosion rate (*R*_corr_) were obtained for carbon steel immersed in aqueous solutions of pH ∼ 5 for the control and for the tested inhibitor systems. They were extracted from the polarization curves shown in Figure 11 and are given in Table 1.

**Table 1:** Electrochemical Parameters for Carbon Steel Maintained for 21 h in Aqueous Solutions of pH ∼ 5 without (Control) and with Inhibitor Systems at 22 °C

sample	*J*_corr_, A/cm^2^	*b*_c_, V	*b*_a_, V	*R*_p_, Ohm	*E*_corr_, V	*R*_corr_, mm/year	IE, %
control	4.791 × 10^–5^	0.200	0.243	5.608 × 10^2^	–0.844	5.964 × 10^–2^	
BPMGLY	3.179 × 10^–6^	0.203	0.312	1.103 × 10^3^	–0.811	3.957 × 10^–2^	84.90
PAIBA	3.138 × 10^–6^	0.225	0.152	6.044 × 10^3^	–0.784	3.906 × 10^–2^	97.32
BPMGLY+Sr^2+^	3.911 × 10^–6^	0.211	0.214	6.382 × 10^3^	–0.805	4.868 × 10^–2^	97.68
PAIBA+Sr^2+^	2.555 × 10^–6^	0.242	0.173	9.049 × 10^3^	–0.721	3.181 × 10^–2^	98.37

The values of *E*_corr_ and *J*_corr_ represent the mean values of three determinations and were calculated from the extrapolation of Tafel lines (anodic and cathodic) placed next to the linearized current regions. The incertitude of parameters lies between 0.83 and 2.29% for *J*_corr_, between 2.18 and 19.22% for *R*_p_, and between 0.44% and 14.85% for *R*_corr_.

The decrease in the corrosion rate is associated with a shift of anodic branches of the polarization curves toward lower current densities with a small positive shift in *E*_corr_. The *J*_corr_ and *R*_corr_ decrease in the presence of all inhibitor systems compared to those for the “control” (no inhibitors) (Table 1). The lowest *J*_corr_ value was observed in the presence of inhibitor systems PAIBA+Sr^2+^ (2.56 × 10^–6^ A·cm^–2^) and PAIBA (3.14 × 10^–6^ A·cm^–2^). The corrosion rate, *R*_corr_, was found to be lower as well for PAIBA+Sr^2+^ (3.18 × 10^–2^ mm/year) and PAIBA (3.91 × 10^–2^ mm/year).

The inhibition efficiency calculated from the corrosion current density data, according to eq 3, also gives good results for PAIBA+Sr^2+^ (∼98%) and PAIBA ∼(97%). The Sr^2+^ ions form insoluble complexes with the carboxy-diphosphonic acids, repair the porous oxide layer, and prevent further corrosion processes.
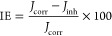
3where IE represents the inhibitory efficiency in %, and *J*_corr_ and *J*_inh_ are the corrosion resistance without and with inhibitors, respectively.

In all cases, a shift in *E*_corr_ to less negative values was observed, indicating coverage of the carbon steel surface with inhibitor molecules and passivation by immersion in aqueous inhibitor solutions through surface complexation. The *E*_corr_ is the lowest in the case of the PAIBA inhibitor and presents a decreasing tendency by adding Sr^2+^. The values of *b*_c_ and *b*_a_ were changed and pointed to different kinetics for the hydrogen evolution reaction and the anodic reaction on the metal surface in the presence of these inhibitors. The high values of Tafel slopes obtained for the specimens exposed to solution with BPMGLY suggest that the corrosion rate calculated for the specimens is underestimated and perhaps is controlled by the diffusion or adsorption phenomena. It was observed that the cathodic slopes (*b*_c_) did not show a defined behavior pattern with respect to inhibitors. It was found that the Sr^2+^/phosphonate inhibitor blends mostly reduced *b*_a_, whereas the “free” phosphonate compounds increased it. The initial adsorption of phosphonic acids at anodic sites and the shape of their adsorbed layers, which involves a layer of positive charges at the metal surface, are thought to be the cause of the decrease in ba induced by these acids when combined with Sr^2+^ inhibitors. The anodic and cathodic processes, however, seem to be primarily inhibited by a hydrophobic layer when the “free” phosphonate compounds are adsorbed on the metal surface. When the two −CH_3_ substituents on the β-carbon are absent, the coadsorption of other ions present in the solution is stimulated. The anionic species present in the system are adsorbed in such a high quantity when BPMGLY is present that the Sr^2+^ ions are essentially neutralized so that these compounds cause an increase in the *b*_a_ values.

The *E*_corr_ values in the presence of inhibitors suggest that the initial step is the adsorption of these molecules on the carbon steel surface. The structure of the formed layer on the metal surface gives the level of protection. Once the inhibitor molecules are adsorbed on the metallic surface, they can be strongly grafted via Fe–O–P(phosphonate) bonds. The metal cations Fe^2+^/Fe^3+^ (from the surface) and/or the added Sr^2+^ cations are expected to form complexes with the phosphonate inhibitor. Such complexes have been reported to be assisting the inhibition effect.^41^

BPMGLY offers an ∼85% corrosion inhibition efficiency (Table 1), and the binary system (BPMGLY with Sr^2+^ and PAIBA with Sr^2+^) demonstrates a nearly quantitative corrosion inhibition efficiency (∼98%). The obtained results indicate lower corrosion protection for BPMGLY and higher for the binary inhibitor system (BPMGLY with Sr^2+^) due to synergistic effects. The results of CP measurements indicate that all “free” inhibitor systems (no Sr^2+^) slow the anodic dissolution of carbon steel and oxygen reduction at the cathodic sites. The effect is more pronounced in the case of carboxy-diphosphonate+Sr^2+^ systems. Similar results were reported in the literature.^39,40^

In general, a shift of the corrosion potential to the positive side and a decrease in the corrosion current density mean that the corrosion reaction of a metal substrate is significantly suppressed by surface modification. Although the corrosion potential of carbon steel is slightly higher than that of carbon steel immersed in inhibitors, there is little difference between the corrosion current densities. Therefore, for the PAIBA+Sr^2+^ blend, a positive impact is brought about by Sr^2+^ ions, which implies the formation of a protective layer on the carbon steel surface because it provides good protection against corrosive species. The increase in polarization resistance is also related to the formation of a protective barrier on the metal surface against the corrosive environment. In the case of BPMGLY and PAIBA inhibitors, the increase in polarization resistance is due to the generation of a hydrophobic layer on the carbon steel surface. When Sr^2+^ is added to the PAIBA inhibitor solution, an increase in polarization resistance is observed because of the formation of a [Sr^2+^···inhibitor] complex next to the hydrophobic organic layer, which leads to a substantial decrease in the corrosion rate.

#### ATR-IR and Imaging of Inhibitive Layers Formed on Carbon Steel Surfaces

**Figure 12:**
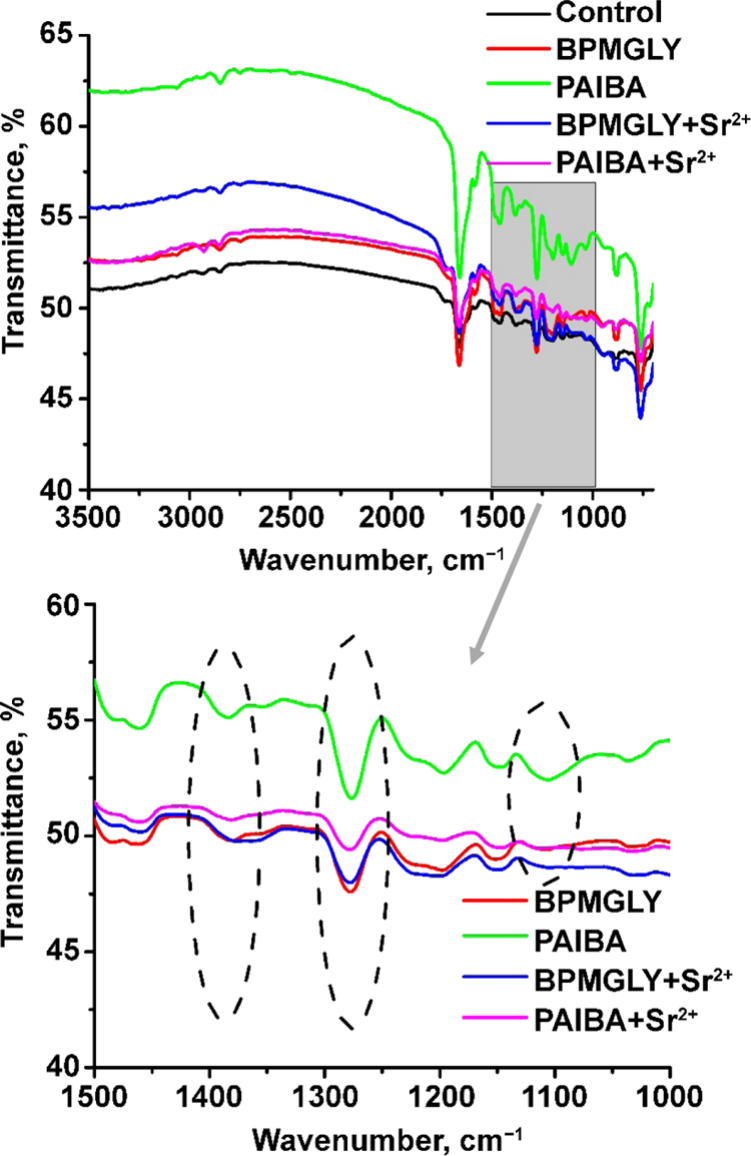
ATR-IR spectra of carbon steel specimens immersed for 120 min in aqueous solutions (pH ∼ 5) in the absence (control) and presence of inhibitors.

The ATR-IR spectra show characteristic bands in the 900–1200 cm^–1^ region assigned to phosphonate group stretching frequencies, as shown in Figure 12. The peaks at 951 and 1100 cm^–1^ are attributed to the symmetric and antisymmetric P–O stretching vibrations from the −PO_3_^–^ moiety. The peaks at 1028 and 1148 cm^–1^ are assigned to the antisymmetric and symmetric stretching of P–O from −PO_3_^2–^. It is observed that the P–O stretching frequency shifts to lower values (from 1080 to 1028 cm^–1^) due to phosphonate coordination to Fe^2+^/Fe^3+^ or Sr^2+^ leading to the formation of [Fe^2+^/Fe^3+^···inhibitor], [Sr^2+^···inhibitor], and/or [Sr^2+^···inhibitor···Fe^2+^/Fe^3+^] complexes on the metal surface (see Figure 12, lower). An increase in the intensity of the peaks corresponding to the antisymmetric stretching vibrations of P–O and P–OH bonds in PO_3_^2–^ is observed in the case of PAIBA and PAIBA+Sr^2+^. This is ascribed to an increase in the number of protonated phosphonate groups in solution.

These bands can be associated with the formation of the Metal–O–P bonds, as phosphonates are coordinated with the species Sr^2+^ or Fe^3+^/Fe^2+^. The metal ions can form metal–phosphonate complexes that cover the metal surface. The band at 1100 cm^–1^ can be attributed to a zwitterionic structure involving intramolecular hydrogen bonding between P–O^–^ and NH^+^.^42^ This band is lower in intensity in the case of inhibitor systems containing Sr^2+^. The bands observed around 1198 and 1278 cm^–1^ correspond to asymmetric stretching vibrations P–O and P=O bond, respectively, from −PO_3_^2–^. The intensity of the band at 1278 cm^–1^, which corresponds to the P=O stretching mode, decreases when Sr^2+^ is present and suggests a tridentate mode of binding to the metal surface and formation of a denser layer (see Figure 12, lower) and corroborates proton dissociation from the phosphonate group, the latter coordinating to the metal ions present. These conclusions are in accordance with the electrochemical data (higher *R*_p_ values).

The symmetric and antisymmetric carboxylate stretching modes are usually found in the 1400–1650 cm^–1^ range. The carboxyl region overlaps with that where C–N and N–H bonds also give rise to absorption bands. These bands are more intense due to deprotonation.^43^

The symmetric carboxylate stretching mode is observed at 1368 cm^–1^, approximately 32 cm^–1^ lower than the typical reported values.^44^ The shift to lower wavenumbers indicates a complexation with the cation present in the solution. The intensity of the antisymmetric carboxylate stretching mode at 1630 cm^–1^ suggests the complexation of the carboxylate group to a metal ion. The weak band at ∼1740 cm^–1^ is likely associated with the antisymmetric vibration characteristic for the −COOH group.^45−47^

The broad band at ∼1460 cm^–1^ was attributed to the bonds C–C, C–H, C–N, and N–H of the aliphatic chains. The intensity of this band is minimal for the “inhibitor+Sr^2+^” systems. The bands at 1584 and 1455 cm^–1^ were assigned to the deformation wagging vibration modes of the protonated amino group. The decrease in intensity in the 1455 cm^–1^ band indicates deprotonation of the −NH^+^ group and is observed for the “inhibitor+Sr^2+^” systems. We have observed this phenomenon before.^38,48^ The spectra for BPMGLY and PAIBA alone show an asymmetry of this band and higher intensity (Figure 12, lower) and suggest that the amino group may still be protonated (at least partially). The possible coordination through the amino group is not clear from ATR-IR spectra due to the overlap with the carboxylic bands in the complex. However, the contribution of the amine group in the “free” inhibitor systems seems to be weak. In the inhibitor+Sr^2+^ systems, it augments the generation of the inhibitive layer. Similar observations were reported in the literature regarding the interaction of glyphosate (*N*-phosphomethylglycine, PMG) or aminomethylphosphonic acid (AMPA).^35,49^ The coordination interactions, involving the electron pairs of N and O (from the carboxylate and phosphonate moieties), between the inhibitor and the metal substrate are facilitated by the addition of Sr^2+^ and, consequently, enhance its adsorption on the carbon steel surface.

The broad band at ∼3350 cm^–1^ was attributed to the −OH stretching vibrations. This absorption band overlaps with the band for N–H and suggests the presence of intermolecular hydrogen bonds.

**Figure 13:**
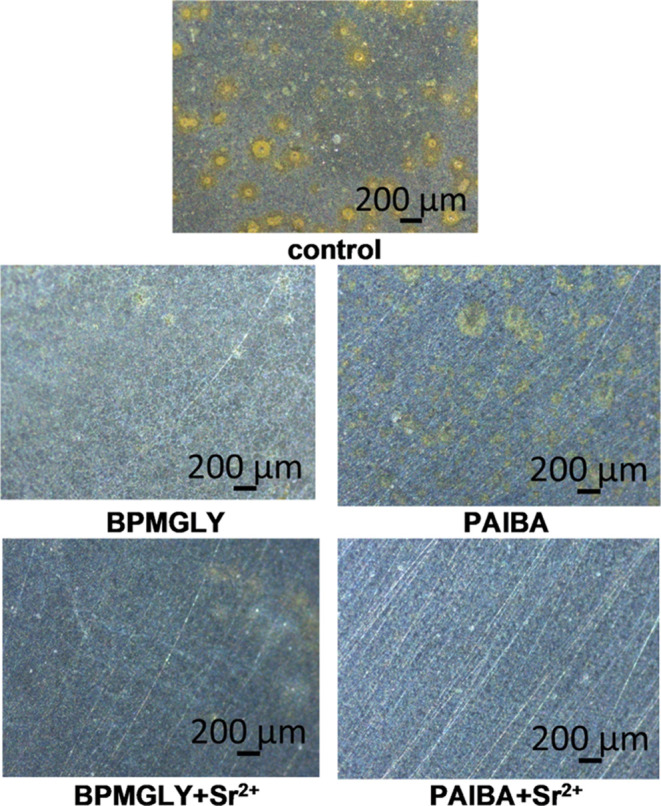
Images of carbon steel specimens immersed for 120 min in aqueous solutions (pH ∼ 5) in the absence (control) and presence of different inhibitor systems, as indicated.

Optical images obtained for the control specimen (no additives were present) revealed general corrosion throughout the entire surface. For the inhibitor systems, the presence of a protective layer on the metal surface was evident. Improved protection was documented for the “inhibitor+Sr^2+^” systems, with optimal results for the “PAIBA+Sr^2+^” blend. Figure 13 displays all of the specimens for comparison.

#### Electrochemical Impedance Spectroscopy (EIS) Measurements and Adsorption

**Figure 14:**
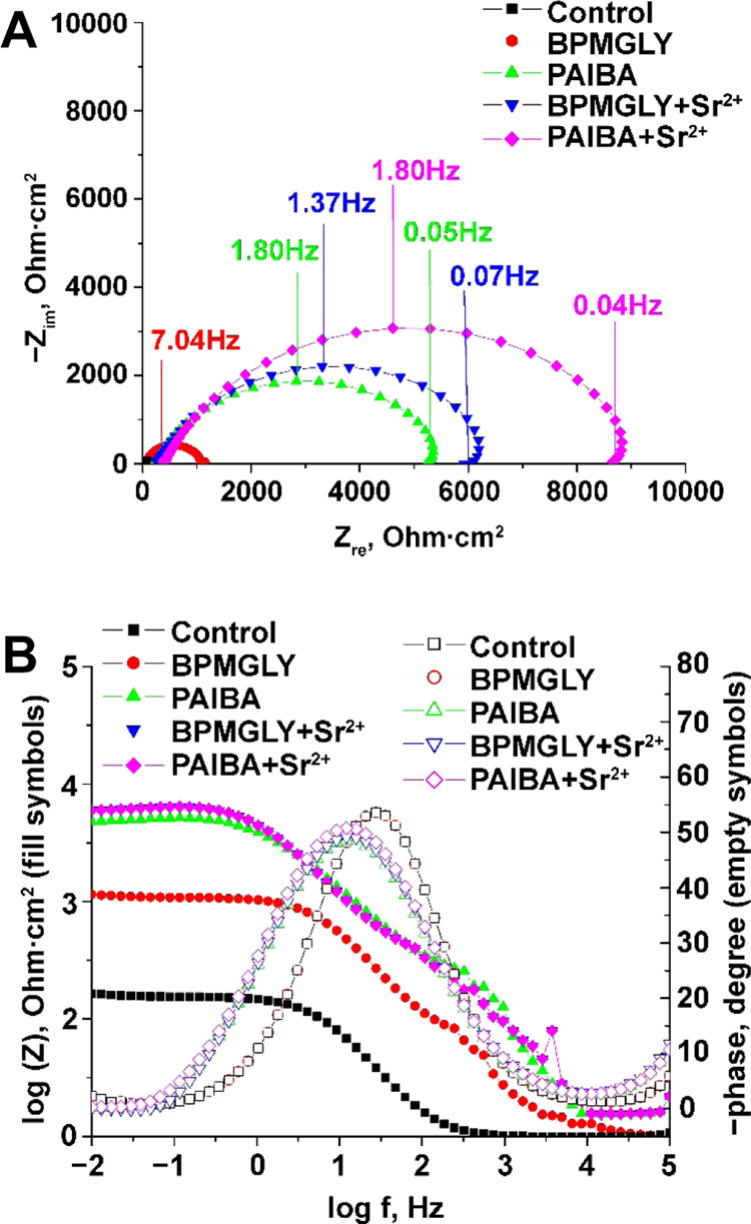
Complex plane Nyquist plots (A) and Bode plots (B) for carbon steel after 120 min immersion in aqueous solutions (pH ∼ 5) in the absence (control) and presence of various inhibitor systems, as indicated.

Impedance spectra are represented as complex impedance diagrams (Nyquist plots) and Bode amplitude and phase angle plots. In the Nyquist graph (Figure 14A), the imaginary component of the impedance is plotted as a function of the real component, whereas the Bode representation (Figure 14B) shows the logarithm of the impedance modulus |*Z*| and phase angle as a function of the logarithm of frequency *f*.

The impedance spectra showed features of an electrode covered with a more-or-less porous layer. In the Nyquist plots, semicircles in the absence and presence of the inhibitors are noted. The diameter of the depressed capacitive loop increased in the presence of an inhibitor. The depressed capacitive loop corresponds to surface heterogeneity as a result of surface roughness, dislocation, or adsorption of the inhibitor molecules. The Nyquist plot reveals a large capacitive loop at high frequencies (HF) related to the charge transfer of the corrosion process and double-layer behavior and an inductive loop at low frequencies (LF) for inhibited systems. This is associated with the relaxation process related to the adsorption and/or incorporation of species (phosphonate polyanions or other charged species) present on and into the iron oxide film. The Bode impedance plots present a large capacitive loop at higher frequency (HF), whereas at intermediate frequency (IF), a small inductive loop is observed, and at low frequency (LF), a capacitive loop is noted. All of these processes overlap and appear in impedance plots as a single loop. The inductive behavior is most likely due to layer stabilization because of the adducts formed between the adsorbed inhibitors and the corrosion products on the metal surface.

**Figure 15:**
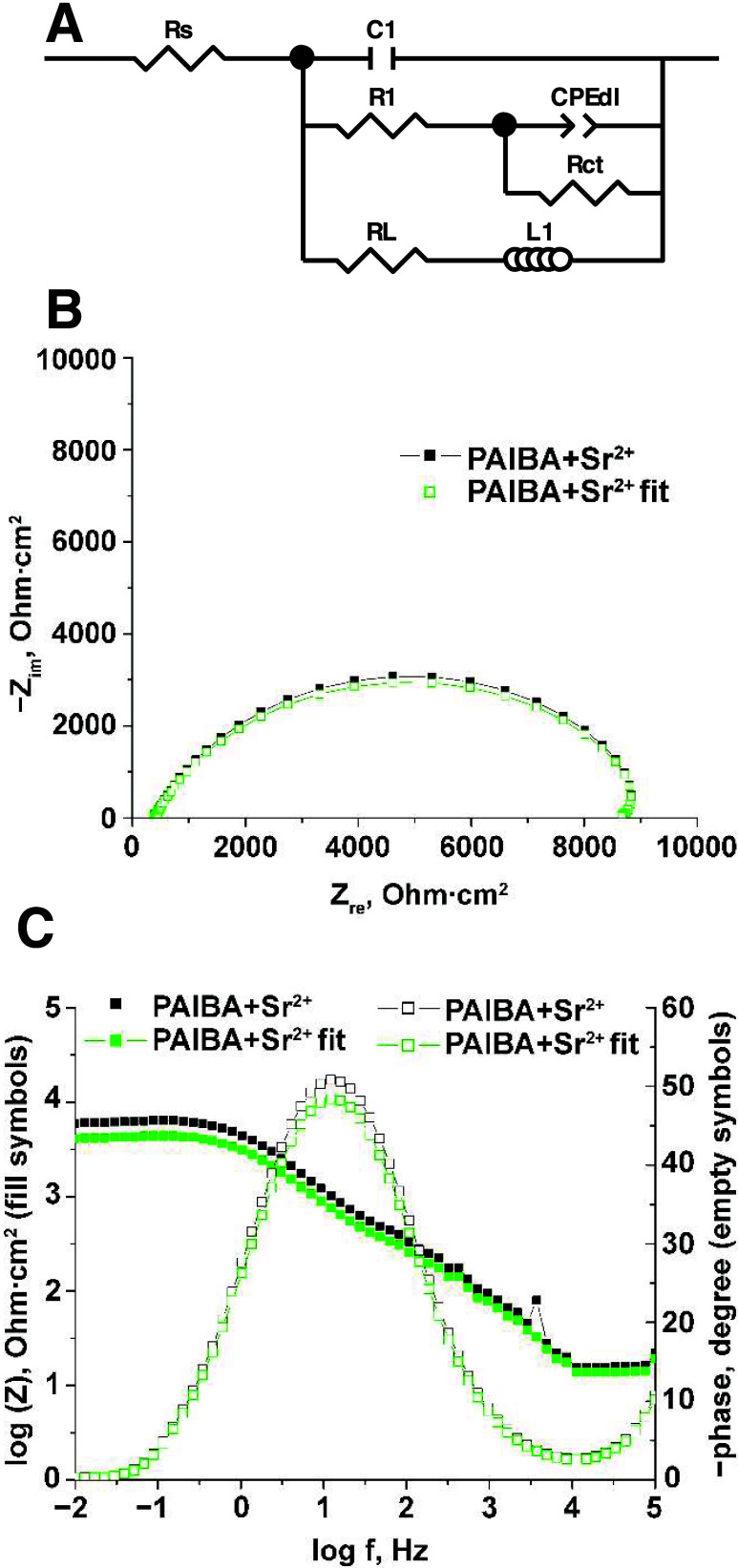
(A) Schematic representation of the equivalent circuit used for modeling the EIS data for carbon steel after 120 min immersion in aqueous solutions pH ∼ 5 without (control) and with inhibitor systems and a representative of example simulation. (B) Nyquist and (C) Bode diagrams with suggested models in the absence and presence of the “PAIBA+Sr^2+^” inhibitor system.

The impedance model for the corrosion was described by the equivalent electric circuit presented in Figure 15A. The notations refer to *R*_s_ as the electrolyte resistance, *C*_1_ in parallel with *R*_1_ as the capacitance and resistance of the inhibitor layer, *C*_dl_ as the double-layer capacitance, and *R*_ct_ as the charge-transfer resistance at high frequencies. The charge-transfer resistance *R*_ct_ is parallel to the electric double-layer capacitance. The inductance *L* is in series with the inductive resistance *R*_L_ and was chosen to fit the low-frequency inductive loop. A representative example of a simulation of Nyquist and Bode diagrams with a proposed model for the PAIBA+Sr^2+^ inhibitor system is illustrated in Figure 15B,C. The best-fitting impedance spectra data presents an error of <8.6%.

The obtained values are summarized in Table 2. The pure double-layer capacitor was replaced by a constant phase element (CPE) to take into account the nonideal behavior, the porosity of the electrode surface, and the pore distribution. CPE-P parameters are noted as CPE-T (T) and CPE-P (φ). If the CPE-P parameter is equal to 1, then the equation is identical to that of a capacitor. The impedance of constant CPE is given by eq 4, and the value of perfect capacitor *C* can be calculated with eq 5.
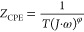
4where 0 < φ < 1 describes the deformation of the circle in the complex plane and *Q* is a constant. If φ = 1, CPE becomes a perfect capacitor. ω is the angular frequency (in rad·s^–1^, with ω = 2π*f*), and *f* is the frequency (in Hz).

**Table 2:** Values of the Electric Circuit Elements for the Electrodes after Immersion for 120 min

specimen	Chi-Sqr	Sum-Sqr	*C*_1_-*T*, F/cm^2^	*R*_1_, Ω·cm^2^	CPE_dl_-*T*, F/cm^2^/sφ^–1^	CPE_dl_-*P*, (φ)	*R*_ct_, Ω·cm^2^	*R*_L_, Ω·cm^2^	*L*_1_, H·cm^2^	IE, %
control	1.35 × 10^–2^	1.51	8.15 × 10^–5^	5.49 × 10^–3^	2.74 × 10^–4^	0.75	153	1212	4432	
BPMGLY	5.08 × 10^–2^	0.57	1.96 × 10^–9^	69.55	4.07 × 10^–5^	0.89	1047	44 711	720.1	84.90
PAIBA	6.09 × 10^–2^	6.83	1.24 × 10^–9^	238.1	3.11 × 10^–5^	0.77	5509	46 102	1.42 × 10^5^	97.32
BPMGLY+Sr^2+^	4.98 × 10^–2^	5.58	1.18 × 10^–9^	269.8	2.70 × 10^–5^	0.79	6530	58 789	88 512	97.68
PAIBA+Sr^2+^	7.24 × 10^–2^	8.11	7.04 × 10^–10^	395.1	1.93 × 10^–5^	0.77	9062	43 907	2.59 × 10^5^	98.37

The *T* parameter is proportional to the capacity of the double layer given by eq 5.
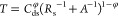
5

*C*_ds_^φ^ = capacity of the double layer, in F

*R*_s_ = solution resistance, in Ω

*A* = charge-transfer resistance, in Ohm

The errors of fit were lower than 3% for CPE_dl_-*P* and *R*_ct_ and lower than 6% for *C*_1_-*T* and *R*_1_. For the inductive loop, the parameters *R*_L_ and *L*_1_ present an error in fitting of around 21%. The relaxation of adsorbed species appears in EIS as a response at low frequencies of EIS and is modeled by parameters *R*_L_ and *L*_1_. These high errors obtained in the modeling process for inductive behavior are due to the low number of points registered at a low frequency. The robustness of the adsorbed layer formed on the surface of the metal depends on the degree of compactness, its effective adhesion on the metal surface, and its thickness. *R*_1_ is a quality measure of the adsorbed inhibitor layer. A low value indicates that the inhibitor layer is thick or incomplete and presents pores or defects. The lowest values for *R*_1_ were obtained for carbon steel immersed in the “free” BPMGLY inhibitor, while the highest value was noted for the “PAIBA+Sr^2+^” inhibitor system (Table 2). BPMGLY compared to PAIBA favors the exposure of carbon steel to the aggressive solution to a greater extent due to its smaller molecular volume, which favors the formation of a porous layer. By adding Sr^2+^, the layer generated on the metal surface is improved. The synergistic action between Sr^2+^ and inhibitors is supported by the higher *R*_1_ values, which reflect the formation of thicker and less porous layers on the metal surface. As a result, these layers can offer superior corrosion resistance. The nonstationary contribution of the adsorbed species on the metal surface, which participates in the entire faradaic process, is generally responsible for the presence of an inductive loop in an impedance diagram. In this scenario, the metal is subjected to an anodic process that generates electrons and the adsorbed species *M*_ads_ (producing a certain degree of surface coverage), which are then subjected to another anodic process that yields additional electrons and metal ions *M*^*n*+^ (*n* represents the total number of lost electrons), leaching into the solution. The synergistic action between Sr^2+^ and inhibitors is supported by the higher *R*_1_ values, which reflect the formation of thicker and less porous layers on the metal surface. As a result, these layers can offer superior corrosion resistance.

The values of *R*_L_ and *L* (Table 2) are indicative of the corrosion mechanism and the durability of protective films generated in an acidic environment. The species that form in the presence of inhibitors are adsorbed on the metal surface; the properties of the adsorbed layer depend on the inhibitor. The phenomena of adsorption and relaxation depend on the charge on the metal surface, the type of interaction between the inhibitors and the metal surface, the chemical structure of the inhibitor itself, and the charge distribution. Compared to PAIBA, BPMGLY favors the exposure of carbon steel to the aggressive solution due to its smaller molecular volume, allowing the formation of a porous layer. In the case of the “BPMGLY+Sr^2+^” inhibitory system, the generated layer on the carbon steel surface is denser than that in the presence of BPMGLY alone but more porous than the layer generated in the case of the “PAIBA+Sr^2+^”. Lower *R*_L_ values were noted when “free” phosphonic acid was present, whereas they increased in the presence of Sr^2+^ and BPMGLY. The fast adsorption of phosphonic acids at anodic sites and the compact shape of their adsorbed layers on the metal surface are thought to be the causes of the decreases in *R*_L_ induced by PAIBA when combined with Sr^2+^. The rapid adsorption suppresses the dissolution of carbon steel and limits corrosion. Therefore, the “complex” between PAIBA and Sr^2+^ seems to play a key role in the formation of stable and compact layers on the metal surface.

The ATR-IR data and images recorded with an optical microscope corroborate the formation of a protective layer in the presence of the inhibitory system with Sr^2+^. Also, the capacities of layer *C*_1_ suggest behavior similar to that suggested by the *R*_1_ values. The capacitance of a parallel-plate capacitor is related to the dielectric constant, the area of the plate, and the separation distance between plates. The change in the capacitance should be due to the change in the layer thickness (since the area of the electrode surface and the dielectric constant of the layers remain unchanged). Thus, an increase in the thickness of the adsorbed layer (a decrease of capacitance value) on the metal surface is observed for the “free” PAIBA inhibitor compared to “free” BPMGLY, but especially in the inhibitory system that also contains Sr^2+^ ions (Table 2).

The high *R*_ct_ value obtained for PAIBA (compared to BPMGLY) shows a decrease in the dissolution of carbon steel due to the blocking of the metal surface with this inhibitor. The inhibitor layer formed in the presence of PAIBA is more compact than that of BPMGLY. A slight increase in the CPE_dl_-*T* values was observed in the presence of the inhibitor. Most likely, this is a result of decreasing surface heterogeneity due to inhibitor adsorption on active adsorption sites. The τ_d_ time constants of the charge-transfer process defined as τ_d_ = *C*_dl_*R*_ct_ are 0.042 s for the control, 0.043 s for “free” BPMGLY, 0.204 s for “free” PAIBA, 0.203 s for “BPMGLY+Sr^2+^”, and 0.204 s for “PAIBA+Sr^2+^”. This increase in τ_d_ values upon the addition of Sr^2+^ in the inhibitor blend shows that the adsorption process occurs faster and the charge transfer is slower.

Due to the experimental errors associated with determining the *R*_L_ and *L*, the information offered by these circuit elements is semiquantitative in nature. The values obtained suggest a different mode of species adsorption, which is also reflected in the different degrees of coverage of the metal surface. It also points out that the adsorbed layer provides the necessary substrate for the formation of the future passive film, a film that, over time, is capable of becoming much denser and thus more protective.^50−52^

The inhibition efficiency (IE) was calculated from the EIS data in eq 6:
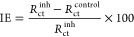
6

where *R*_ct_^inh^ is the charge-transfer resistance for the electrode in the presence of the inhibitor and *R*_ct_^control^ is the charge-transfer resistance for the electrode without an inhibitor.

The % inhibition efficiency reveals that optimal results are obtained with the “PAIBA+Sr^2+^” system (98.37%). The calculated values are close to those obtained from the polarization data. The aforementioned inhibition efficiency is comparable to that reported in the literature for BPMGLY on carbon steel, with or without bivalent cations,^53^ or amino-*tris*(methylenephosphonic) acid (ATMP) with Cu^2+^, Mn^2+^, Ca^2+^, or Zn^2+^.^54^

Based on the surface coverage (θ) obtained from EIS and calculated with eq 7, the adsorption isotherm was determined by taking into account the best correlation coefficient.^55^
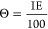
7

The adsorption of the inhibitor molecules on carbon steel is best described by the Langmuir adsorption isotherm (eq 8). The correlation coefficient (*R*^2^) is close to unity and indicates the existence of molecular adsorption in the adsorbed layer.
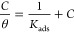
8

where θ is the surface coverage, *C* = *C*_inhibitor_ is the inhibitor concentration, and *K*_ads_ is the adsorption equilibrium constant.

**Figure 16:**
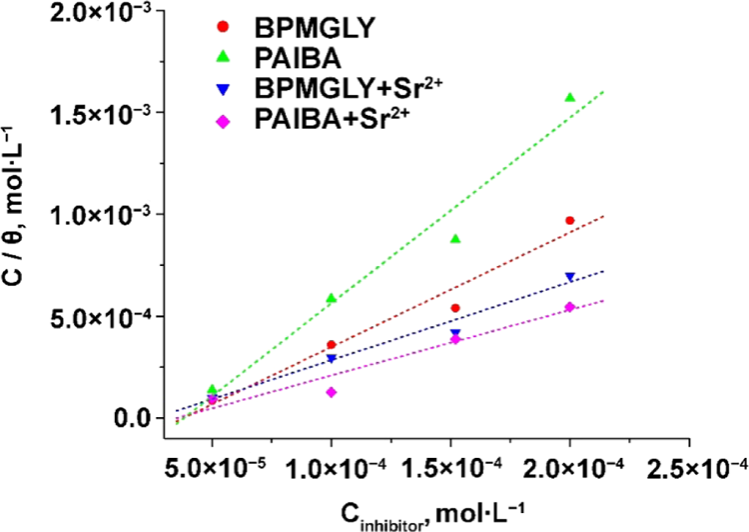
Langmuir isotherm plots for all tested inhibitor systems, as indicated.

The linear Langmuir plots (*C*/θ versus *C*) are shown in Figure 16.

The free energy values of the adsorption (Δ*G*°_ads_) were calculated by eq 8a and are listed in Table 3.
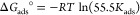
8a

**Table 3:** Adsorption Data for the Inhibitor Systems on Carbon Steel at pH ∼ 5

inhibitor system				
	BMPGLY	PAIBA	BPMGLY+Sr^2+^	PAIBA+Sr^2+^
*K*_ads_ (mol^–1^)	2846.262	4607.488	8600.449	9824.148
Δ*G*°_ads_ (J/mol)	–29 656.9	–30 850.3	–32 396.6	–32 726.2
*R*_L_	0.78	0.68	0.54	0.50

where *R* is the universal gas constant (8.314 J·mol^–1^·K^–1^), *T* is the absolute temperature (in Kelvin), *K*_ads_ is the adsorption equilibrium constant, Δ*G*_ads_^o^ is the standard free energy of adsorption, and 55.5 is the concentration of water in the solution in mol·dm^–3^.

*K*_ads_ is characteristic of the nature of inhibitor adsorption onto the metal surface. More effective adsorption results in higher *K*_ads_ values. The negative values of Δ*G*_ads_^o^ and the high values of the obtained *K*_ads_ also indicate that the adsorption process in the studied cases is spontaneous and leads to the formation of an inhibitory layer on the metal surface. For the inhibitor systems studied, the Δ*G*°_ads_ values range from −29.66 to −32.40 kJ·mol^–1^.

In general, the lower values of Δ*G*°_ads_ (or closer to −20 kJ·mol^–1^) point to a physisorption process, while more negative than −40 kJ·mol^–1^ values involve chemisorption (i.e., chemical bond formation).^56^ For the inhibitor systems studied, the values of Δ*G*°_ads_ indicate that the adsorption of the inhibitors on the metal surface occurs by a combined process, both physisorption and chemisorption.^57^ The most effective absorption occurs in the “PAIBA+Sr^2+^” inhibitory system and is the result of concurrent physical and chemical adsorption. The weaker adsorption in the case of the BPMGLY inhibitor is mainly due to the predominance of physical interactions. The synergistic action in the “PAIBA+Sr^2+^” system is also demonstrated by the adsorption data. This inhibitory system presents a high capacity to interact not only physically but also chemically with the metal surface.

The dimensionless separation factor *R*_L_ calculated for the inhibitors studied at 0.1 mmol·L^–1^ concentration (eq 9) shows that the adsorption process is favorable, as the obtained values are <1 (the process is favored when 0 < *R*_L_ < 1).
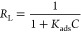
9

These results agree with the reported adsorption behavior of phosphonic acids on the metal surface and display Langmuir adsorption isotherms.^38,48,58−60^

A plausible mechanism for corrosion inhibition based on the present results by considering the synergistic effect between the inhibitors and Sr^2+^ is presented below. The first step is the formation of Fe^2+^ ions at the anode sites (eq 10):
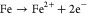
10Under the action of the oxygen available in the aqueous solution, the oxidation of Fe^2+^ to Fe^3+^ then takes place (eq 11):
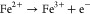
11The corresponding reduction reactions at the cathodic sites in the acidic medium are (eqs 12 and 13):
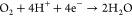
12
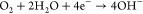
13

**Figure 17:**
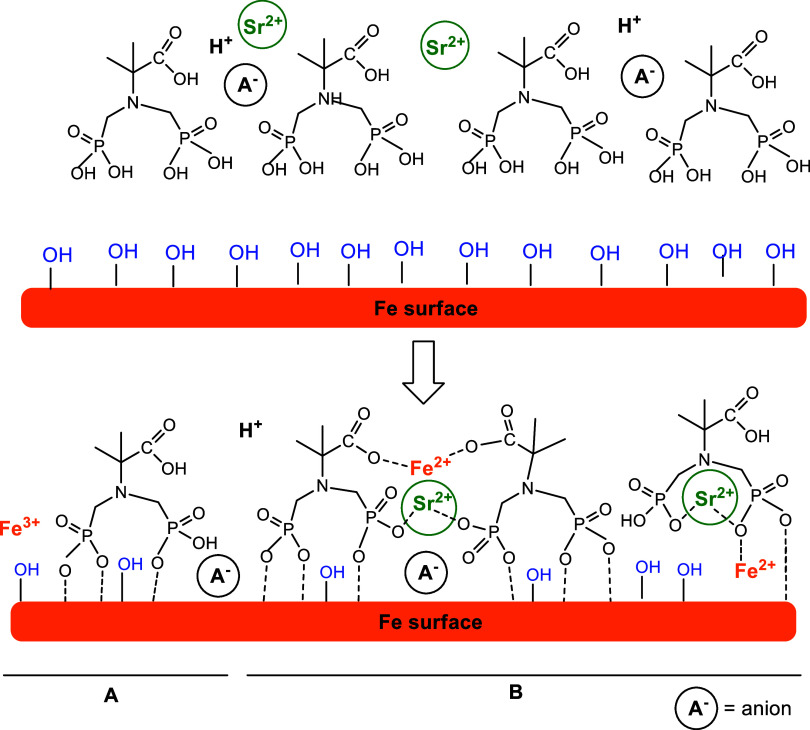
Schematic representation of (A) immobilization and (B) coordination-induced deposition of the PAIBA phosphonate inhibitor on the carbon steel substrate forming [Sr^2+^···inhibitor] and [Sr^2+^···inhibitor···Fe^2+^/Fe^3+^]_ads_ “complexes”.

The formed species combine at the anodic and cathodic areas, resulting in the formation of oxides and hydroxides (e.g., FeOOH, Fe(OH)_2_, γ-Fe_2_O_3_, etc.) on the metal surface. The oxidation of metallic iron (Fe^0^) to ferric iron (Fe^3+^) on the electrode surface follows a two-step process (Fe^0^ → Fe^2+^ → Fe^3+^). Then, near the anode, Fe^3+^ reacts with the OH^–^ formed at the cathode, producing Fe(OH)_3_ or a mixture of Fe(OH)_3_ and FeOOH. The iron hydroxides formed at the anode or in the bulk solution migrate to the cathode, where they are reduced to form Fe_3_O_4_. The mechanism for the oxidation of Fe to Fe^2+^ is complex since the experimental results obtained by EIS show inhibitor-dependent differences in the inductive and capacitive loops, which can be associated with the adsorption phenomena of the intermediate species. The inhibitor molecules are first anchored covalently on the carbon steel substrate through the electrostatic and hydrogen-bonding interactions. These involve oxygen atoms from P=O, P–OH, and hydroxyl groups on the iron substrate forming P–O···H–O–Fe bonds, leading to the (proposed) complex [Fe^2+^/Fe^3+^···inhibitor] (Figure 17a). The inhibitor binds more strongly to the metallic surface through Fe–O–P(phosphonate) bonds that form by a dehydration reaction.

Free phosphonic or carboxylic groups can be adsorbed onto the corrosion products, thus sealing the pores in the film. Upon addition of the inhibitor and Sr^2+^ to the aqueous solution, the inhibitor reacts with Sr^2+^ and forms a soluble [Sr^2+^···inhibitor] complex (Figure 17b). This complex can then diffuse from the solution toward the metal surface. There, it reacts with ions present on the metal surface and forms a new complex of proposed composition [Sr^2+^···inhibitor···Fe^2+^/Fe^3+^]. The newly formed complex covers the anodic sites (eq 14) and endows anodic protection.
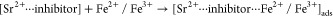
14

At the cathodic sites, Sr^2+^ ions present in the solution diffuse near the metal surface and form Sr(OH)_2_ that covers and protects the cathodic sites (eq 15).
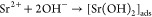
15

Thus, the inhibitor and Sr^2+^ bind more strongly and lead to more efficient packing on the surface. A similar mechanism was proposed for propyl phosphonic acid (PPA) as a corrosion inhibitor in association with a divalent cation such as Zn^2+^.^61^ By blending Ca^2+^, Mg^2+^, or Ba^2+^ with PAIBA, less compact films are generated on the electrode surface; hence, the access of water molecules and aggressive species to the electrode surface is uninhibited. A possible explanation for the enhanced corrosion inhibition observed for the “PAIBA+Sr^2+^” versus the “BPMGLY+Sr^2+^” system can be based on the following arguments. The initial adsorption at the anodic sites of PAIBA and BPMGLY inhibitors when combined with Sr^2+^ ions leads to the formation of adsorbed layers, but these are different. In the case of BPMGLY, without the two −CH_3_ substituents on the β-carbon, the coadsorption of ions from the solution is higher, and the synergic action of Sr^2+^ ions is partially compromised. By adding Sr^2+^ to PAIBA, the generated films are more compact and restrict access to the electrode surface. This is corroborated by the higher polarization resistance values.

Since organic phosphonic acids have low toxicity, high stability, and corrosion inhibition qualities, their application in protecting carbon steel has been documented in the literature. The inhibition efficiency of various compounds, mostly phosphonates, as corrosion inhibitors for iron is compiled in Table 4, along with our present findings.

**Table 4:** Inhibition Efficiencies of Different Compounds and Phosphonates Cited in the Literature as Corrosion Inhibitors for Iron in Various Solutions

inhibitor system	electrode/electrolyte	inhibition efficiency, %	refs
*N,N*-*bis*(phosphonomethyl)glycine 20 ppm, Zn^2+^ 30 ppm, Na_2_WO_4_ 200 ppm	carbon steel/200 ppm of NaCl	95.93	(40)
*N*-phosphonomethylglycine (NPMG)/Zn^2+^ 1:1	carbon steel/0.5 M NaClO_4_, pH ∼ 7	corrosion rate reduced by 20%	(41)
20 ppm *N*-phosphonomethylglycine, 30 ppm Zn^2+^, 25 ppm ascorbic acid	carbon steel/pH = 5–11	94	(47)
diethylenetriaminepentamethylenephosphonic acid (DTPMP) 250 ppm + Ni^2+^ (50 ppm)	carbon steel/sea water	70	(64)
1-hydroxyethane-1,1-diphosphonic acid (HEDP) 200 ppm + Na_2_WO_4_ 50 ppm + Zn^2+^ 10 ppm	pH = 8.38/Cl^–^ 665 ppm	98	(65)
octylamino-di(methylenephosphonate) C8-D/4.6 mM	3.5% NaCl	93.89	(48)
Sr^2+^/DPMG (2:1)	Armco iron/0.1 M NaClO_4_	90	(53)
amino-tris(methylenephosphonate)/Zn^2+^ 1:1	carbon steel/HCl 1M	85.08	(54)
5-bis(4-methoxyphenyl)-1,3,4-oxadiazole, 8 × 10^–4^ M	carbon steel/0.5 M H_2_ SO_4_	83	(55)
octylphosphate, 50 ppm	iron/0.5 M H_2_ SO_4_	88	(58)
ethyl hydrogen [(3-methoxyphenyl)(methylamino)methyl]phosphonate, 10^–3^ M	mild steel/in 1 M HCl	75.58	(59)
	0.5 M H_2_SO_4_	86.64	
PAIBA/Sr^2+^, 1:1 molar ratio, 5 mM	carbon steel/pH ∼ 5	98.37	this work

The present results imply that the studied phosphonic acids are suitable candidates for iron protection since they demonstrate that a protective coating can be generated on the metal surface, delaying the corrosion reaction. These multidimensional hybrid metal phosphonate inhibitor systems offer an improved corrosion inhibition efficiency. The latter is dependent on alkaline-earth metal ions already present in the process water. Τheir abundance depends on the specific application; for example, in the oilfield sector, Ba^2+^ ions are abundant. Phosphonate additives are widely used as scale inhibitors to combat crystallization and subsequent deposition of mineral scales. Hence, with the proper choice of phosphonate (see, for example, Table 4 for a number of commercially available compounds), both scale and corrosion inhibition could be achieved. This is very important for the water industry, and efforts to discover, evaluate, and apply such “dual” action inhibitors are underway in our laboratory.

## Conclusions

This work is part of our continuing efforts to map metal phosphonate chemistry from a synthetic and structural point of view toward surface chemistry and related applications, particularly corrosion protection,^8,9,11,12^ and crystal growth inhibition.^62,63^ Herein, we reported a family of four new hybrid metal phosphonate materials that are constructed from alkaline-earth metal ions (Mg^2+^, Ca^2+^, and Sr^2+^) and the carboxy-diphosphonate linker PAIBA. The structural types of the obtained compounds range from a 0D dinuclear complex (Mg-PAIBA) to 3D (Ca-PAIBA) and 1D (Sr-PAIBA and Sr-Na-PAIBA) coordination polymers. These new compounds were also crystallographically and topologically studied and classified, revealing a variety of underlying networks with 6,10T9, **unc**, SP 1-periodic net (4,4)(0,2), and unique topologies.

The behavior of steel surfaces in mildly acidic solutions (pH ∼ 5) containing either BPMGLY and PAIBA alone or in combination with Sr^2+^ was studied using electrochemical methods. The polarization results show a corrosion inhibition efficiency of ∼98% for the “PAIBA+Sr^2+^” system at a molar ratio of 1:1. The inhibitory efficiency calculated from the EIS data also confirms the optimal results for this system.

The presence of inhibitors causes a reduction in *J*_corr_ and *R*_corr_. The lowest *J*_corr_ and *R*_corr_ values were observed with the “PAIBA+Sr^2+^” blend. The EIS measurements show that the time constant τ_d_ of the charge-transfer process decreases in the presence of Sr^2+^. Adsorption of inhibitor molecules on carbon steel follows a Langmuir isotherm. The adsorption values of Δ*G*°_ads_ in the presence of inhibitors range from −29.66 to −32.40 kJ·mol^–1^. The strongest absorption on the metal surface occurs in the “PAIBA+Sr^2+^” inhibitory system and is the result of a combination of physical and chemical adsorption. In the case of the BPMGLY inhibitor, the adsorption observed is mainly due to physical interactions. The proposed mechanism involves a more efficient packing of the inhibitor on the metallic surface in the presence of Sr^2+^ due to the formation of a [Sr^2+^···inhibitor···Fe^2+^/Fe^3+^] complex that is capable of covering the anodic sites, while Sr(OH)_2_ species interact with the cathodic sites, contributing to surface protection.

The present work reveals that by broadening the family of carboxyphosphonate-based coordination networks and showing that these compounds (the metal-PAIBA and the related BPMGLY systems) can act as attractive hybrid coatings with anticorrosion performance, this study contributes to the development of new functional materials for corrosion prevention.

## Data Availability

Data for the research described in this article are available from the corresponding author upon request.

## Supporting Information Available

Tables with crystallographic data, corrosion rates, and % inhibition for PAIBA- and BPMGLY-containing systems; NMR (^1^H, ^13^C, ^31^P), ATR-IR, and TGA data; and optical images of corrosion specimens and graphs with % inhibition efficiencies (PDF)
