# Supplementary material for: Multidimensional Hybrid Metal Phosphonate Coordination Networks as Synergistic Anticorrosion Coatings
Source: Inorg Chem. 2024 Aug 12;63(34):16018–36. doi: 10.1021/acs.inorgchem.4c02545 (PMC11351182; doi:10.1021/acs.inorgchem.4c02545)
Supplement: Supplementary file 1 — ic4c02545_si_001.pdf [file ic4c02545_si_001.pdf]

Supporting Information

For

Multidimensional Hybrid Metal Phosphonate Coordination Networks as Synergistic Anti-Corrosion Coatings

Apostolos Fanourgiakis,[a] *Elpiniki Chachlaki,*[a] *Nicoleta Plesu,*[b] *Duane Choquesillo-Lazarte,*[c] *Alexander M. Kirillov,*[d] *and Konstantinos D. Demadis*[*a]

[a] Crystal Engineering, Growth and Design Laboratory, Department of Chemistry, University of Crete, Voutes Campus, Heraklion, Crete, GR-71003, Greece

[b] Institute of Chemistry Timisoara of Romanian Academy, 300223 Timisoara, Romania

[c] Laboratorio de Estudios Cristalográficos, IACT, CSIC-Universidad de Granada, Granada-18100, Spain

[d] Centro de Química Estrutural, Institute of Molecular Sciences, Departamento de Engenharia Química, Instituto Superior Técnico, Universidade de Lisboa, Av. Rovisco Pais, 1049-001, Lisbon, Portugal

*Email: demadis@uoc.gr

Table of contents

Tables

Table S1. Crystallographic data for the reported materials.
Table S2. Corrosion rates (mm/y) and % inhibition of all systems containing BPMGLY.
Table S3. Corrosion rates (mm/y) and % inhibition of all systems containing PAIBA.

Figures

Figure S1. ^{1}H NMR spectrum of PAIBA.
Figure S2. ^{31}P NMR spectrum of PAIBA.
Figure S3. ^{13}C NMR spectrum of PAIBA.
Figure S4. ^{1}H NMR spectrum of BPMGLY.
Figure S5. ^{31}P NMR spectrum of BPMGLY.
Figure S6. ^{13}C NMR spectrum of BPMGLY.
Figure S7. Comparative ATR-IR spectra of M^{2+}-PAIBA compounds (M^{2+} = Mg^{2+}, Ca^{2+}, Sr^{2+}).
Figure S8. Comparative powder X-ray diffraction diagrams [calculated (red) vs. measured (blue)] of all M^{2+}-PAIBA compounds (M^{2+} = Mg^{2+}, Ca^{2+}, Sr^{2+}).
Figure S9. TGA traces of all M^{2+}-PAIBA compounds (M^{2+} = Mg^{2+}, Ca^{2+}, Sr^{2+}).
Figure S10. Optical images of the carbon steel surfaces after immersion in the presence of BPMGLY inhibitor and its combination with alkaline earth metal ions, at pH 4.0 and at concentrations 0.1 mM, 0.5 mM and 1.0 mM.
Figure S11. Optical images of the carbon steel surfaces after immersion in the presence of BPMGLY inhibitor and its combination with alkaline earth metal ions, at pH 5.0 and at concentrations 0.1 mM, 0.5 mM and 1.0 mM.
Figure S12. Optical images of the carbon steel surfaces after immersion in the presence of BPMGLY inhibitor and its combination with alkaline earth metal ions, at pH 6.0 and at concentrations 0.1 mM, 0.5 mM and 1.0 mM.
Figure S13. Optical images of the carbon steel surfaces after immersion in the presence of PAIBA inhibitor and its combination with alkaline earth metal ions, at pH 4.0 and at concentrations 0.1 mM, 0.5 mM and 1.0 mM.
Figure S14. Optical images of the carbon steel surfaces after immersion in the presence of PAIBA inhibitor and its combination with alkaline earth metal ions, at pH 5.0 and at concentrations 0.1 mM, 0.5 mM and 1.0 mM.
Figure S15. Optical images of the carbon steel surfaces after immersion in the presence of PAIBA inhibitor and its combination with alkaline earth metal ions, at pH 6.0 and at concentrations 0.1 mM, 0.5 mM and 1.0 mM.
Figure S16. Comparative inhibition efficiency (%) data for the metal-PAIBA and metal-BPMGLY inhibitor systems, at pH = 4.0 and at concentrations 0.1 mM, 0.5 mM and 1.0 mM.
Figure S17. Comparative inhibition efficiency (%) data for the metal-PAIBA and metal-BPMGLY inhibitor systems, at pH = 5.0 and at concentrations 0.1 mM, 0.5 mM and 1.0 mM.
Figure S18. Comparative inhibition efficiency (%) data for the metal-PAIBA and metal-BPMGLY inhibitor systems, at pH = 6.0 and at concentrations 0.1 mM, 0.5 mM and 1.0 mM.

Figure S19. Optical images of the carbon steel surfaces used for the EDS studies, after immersion for ~ 10 days in the absence of inhibitors (control), and in the presence of inhibitor as shown, at pH 6.0 at the concentration 1.0 mM of PAIBA, BPMGLY, and Sr^{2+}. All specimens show the expected presence of Fe. Specimens treated only with phosphonate show the presence of P, whereas those treated with Sr^{2+} and phosphonate show the presence of both Sr and P.

Table S1. Crystallographic data for the reported materials.

	**Mg-PAIBA** **$Mg_2(PAIBA)_2(H_2O)_6 \cdot 6H_2O$**	**Ca-PAIBA** **$Ca(PAIBA)(H_2O) \cdot 2H_2O$**	**Sr-PAIBA** **$Sr(PAIBA)(H_2O) \cdot 4H_2O$**	**Sr-Na-PAIBA** **$Sr_2Na_{0.5}(PAIBA)_2(H_2O) \cdot 6H_2O$**
**Abbreviation**	Mg-PAIBA	Ca-PAIBA	Sr-PAIBA	Sr-Na-PAIBA
**Formula**	$C_{12}H_{50}Mg_2N_2O_{28}P_4$	$C_6H_{19}CaNO_{11}P_2$	$C_6H_{23}SrNO_{13}P_2$	$C_{24}H_{78}N_4\ Sr_4NaO_{46}P_8$
**FW (g·mol^{-1})**	843.04	383.24	466.81	1780.13
**Crystal system**	Triclinic	Orthorhombic	Monoclinic	Monoclinic
**Space group**	$P\bar{1}$	$P2_12_12_1$	$P2_1/c$	C2
a **(Å)**	9.505(5)	7.1401(4)	14.0041(7)	30.8338(14)
b **(Å)**	9.667(5)	13.8895(8)	7.2181(3)	7.3147(3)
c **(Å)**	10.494(6)	14.2841(8)	16.9336(7)	13.9216(7)
**α (deg)**	87.834(18)	90.000	90.000	90.000
**β (deg)**	87.781(14)	90.000	91.113(3)	93.349(2)
**γ (deg)**	68.244(15)	90.000	90.000	90.000
**V (Å^3)**	894.6(8)	1416.59(14)	1711.38(13)	3134.5(2)
**Crystal size (mm)**	0.14 × 0.13 × 0.10	0.14 × 0.13 × 0.10	0.11 × 0.11 × 0.10	0.14 × 0.12 × 0.12
Z	2	4	4	2
**D_{calc} (g·cm^{-3})**	1.565	1.797	1.812	1.886
**Temperature (K)**	100(2)	298(2)	298(2)	298(2)
**θ range (deg)**	2.269−26.428	2.85−27.64	3.156−66.458	2.862− 27.645
**Reflns collected**	32207	9947	13888	25365
**Obsd reflns [$I > 2\sigma(I)$]**	3674	3296	3010	9953
**Data/restraints /parameters**	2800/0/233	2995/0/194	2621/0/212	6409/1/398

**GOF (on F^2)**	1.049	1.074	1.148	1.017
**R factor [$I > 2\sigma(I)$]**	R_1 = 0.0422<br>wR_2 = 0.0837	R_1 = 0.0437<br>wR_2 = 0.1073	R_1 = 0.0499<br>wR_2 = 0.1229	R_1 = 0.0312<br>wR_2 = 0.0691
**R factor (all data)**	R_1 = 0.0677<br>wR_2 = 0.0924	R_1 = 0.0503<br>wR_2 = 0.1104	R_1 = 0.0571<br>wR_2 = 0.1264	R_1 = 0.0381<br>wR_2 = 0.0714
**Maximum peak/hole (e·Å^{-3})**	0.563/−0. 595	0.377/−0.576	0.900/−-0.829	0.083/− 0.756
**CCDC number**	2314234	2314235	2314236	2314237

1. Characterization of the carboxy-diphosphonate ligands

The NMR spectra were collected at a Bruker DPX-300 spectrometer in D_2O. The corresponding ^{1}H (NS: 36), DEPT135 ^{13}C (NS: 800) and ^{31}P (NS: 36). NMR spectra of PAIBA and BPMGLY are presented in the following Figures as indicated.

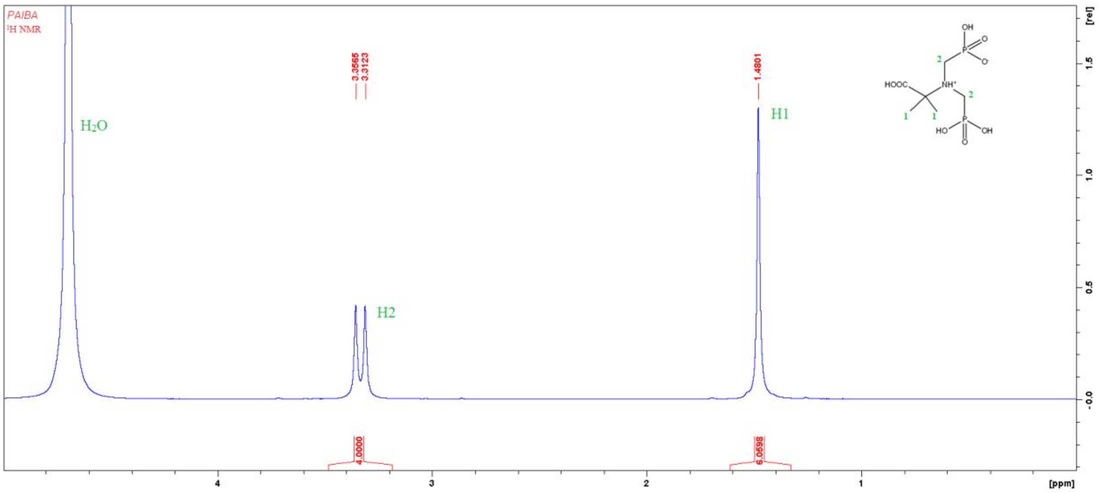

Figure S1. ^{1}H NMR spectrum of PAIBA.

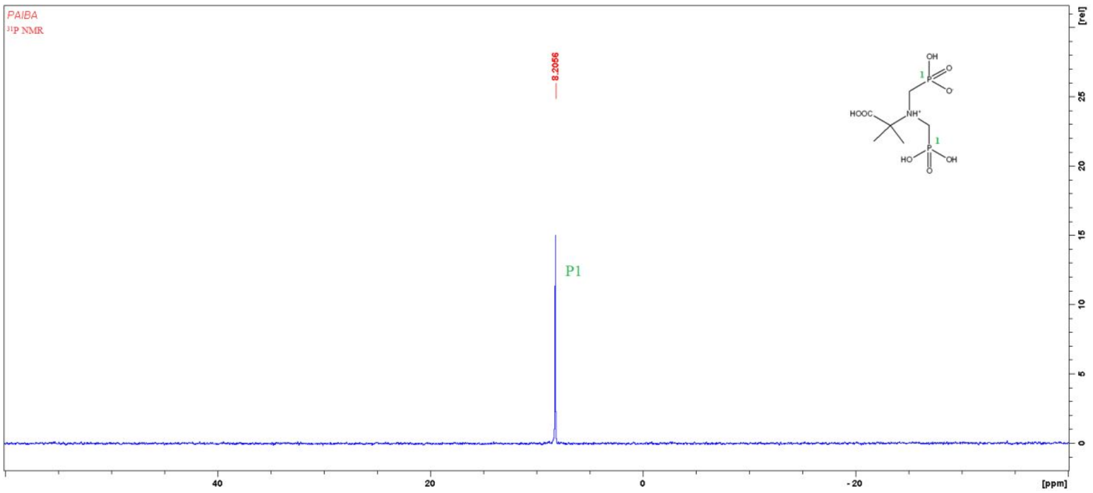

Figure S2. ^{31}P NMR spectrum of PAIBA.

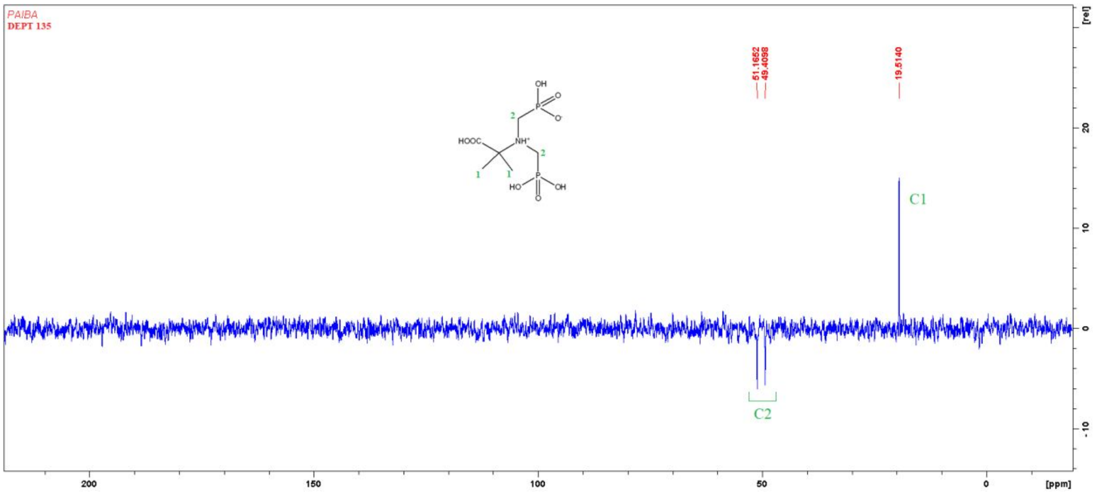

Figure S3. ^{13}C NMR spectrum of PAIBA.

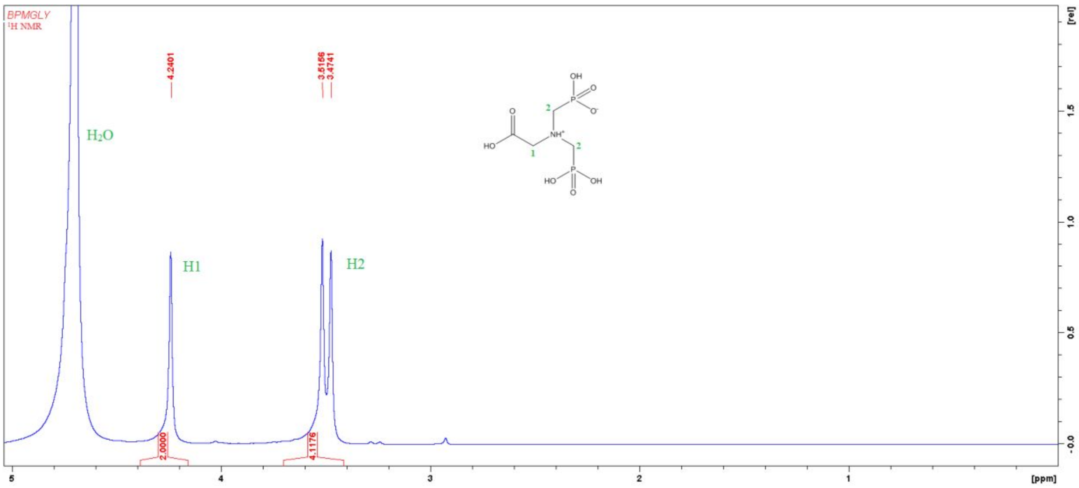

Figure S4. ^{1}H NMR spectrum of BPMGLY.

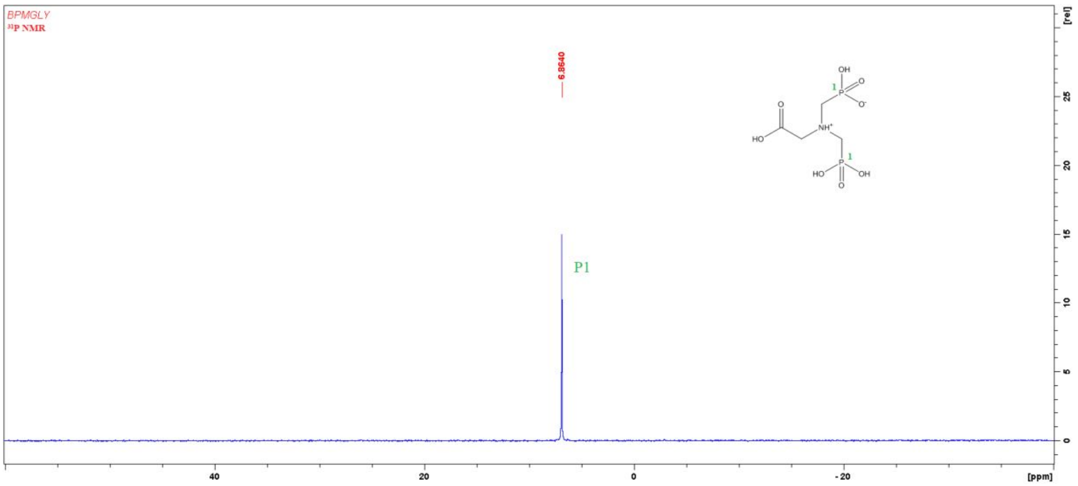

Figure S5. ^{31}P NMR spectrum of BPMGLY.

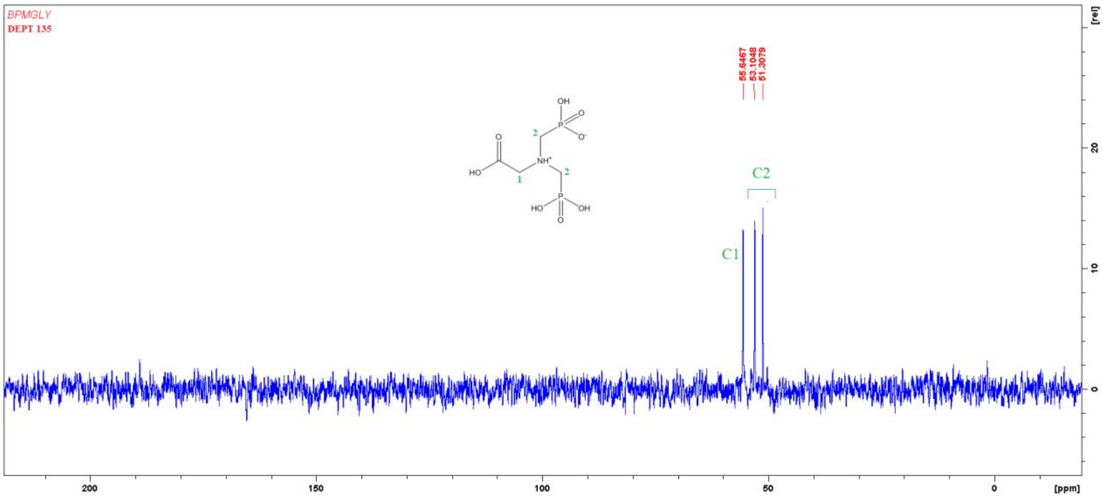

Figure S6. ^{13}C NMR spectrum of BPMGLY.

2. Characterization of M^{2+}-PAIBA compounds

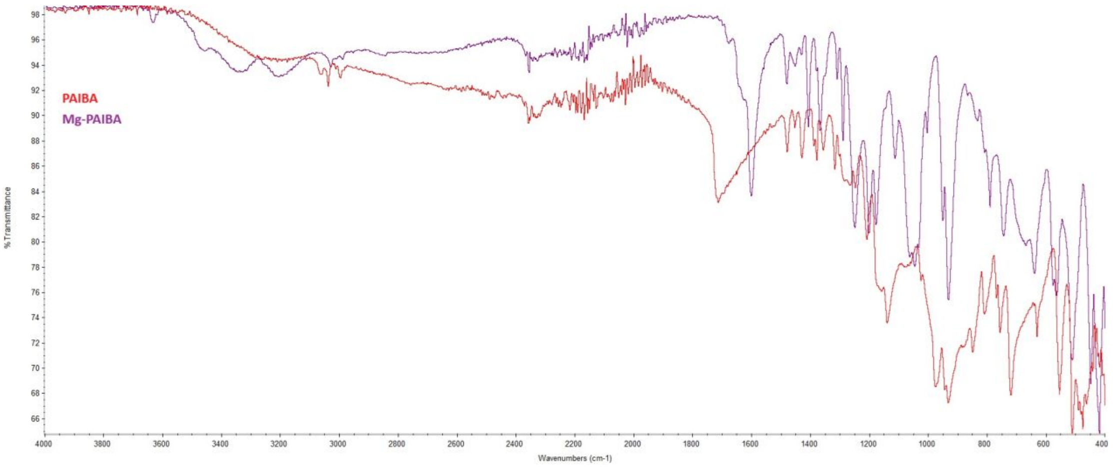

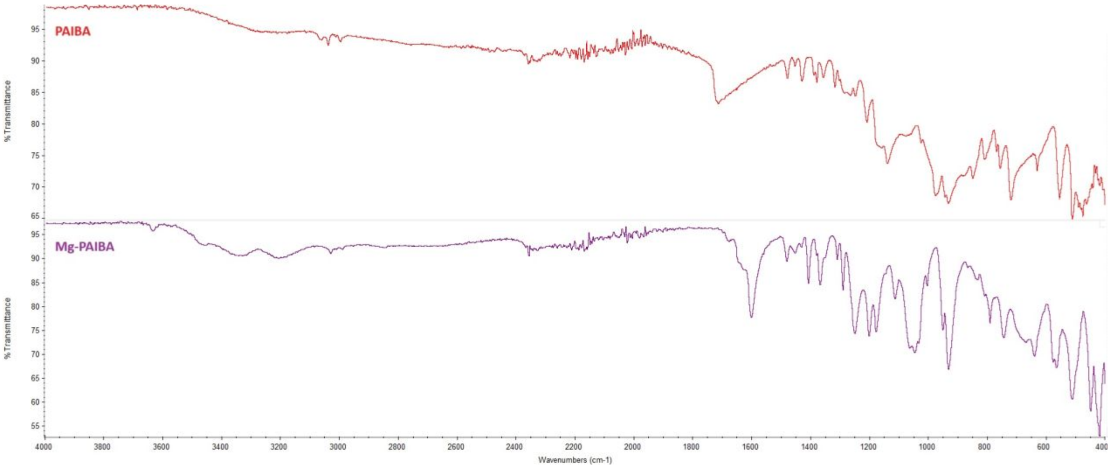

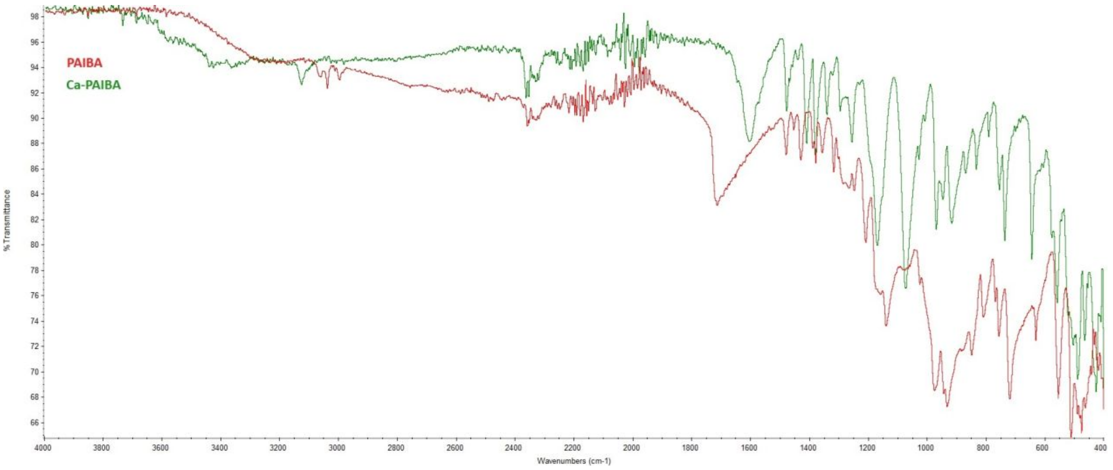

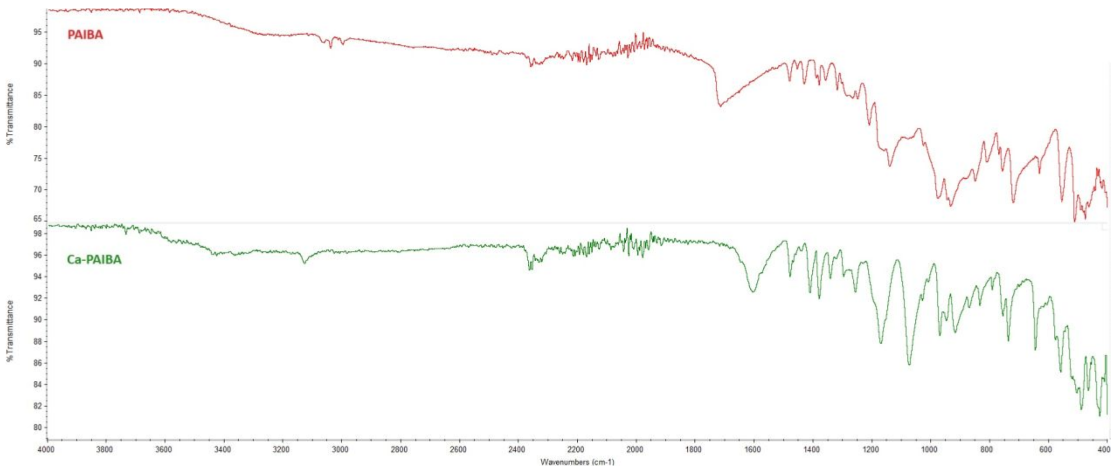
PAIBA
Ca-PAIBA
% Transmittance
% Transmittance
Wavenumbers (cm-1)

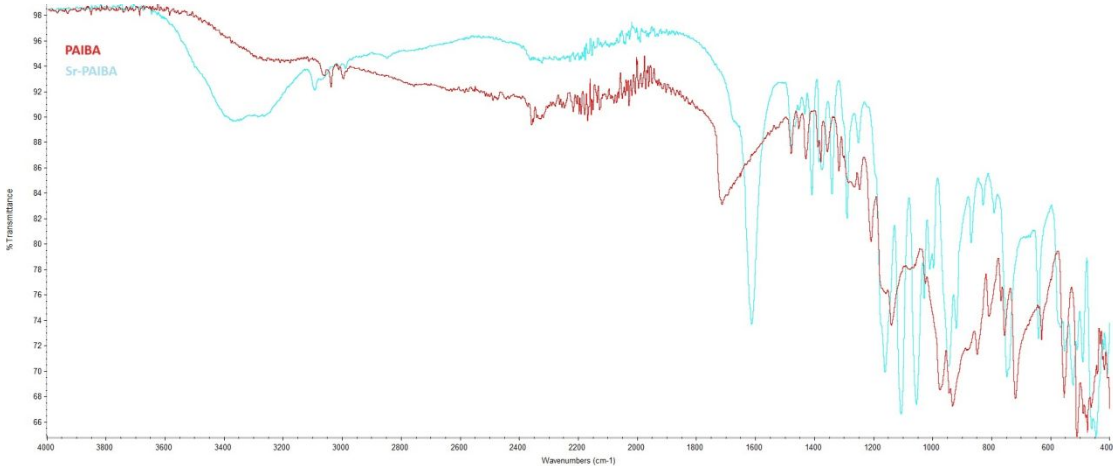
PAIBA
Sr-PAIBA
% Transmittance
Wavenumbers (cm-1)

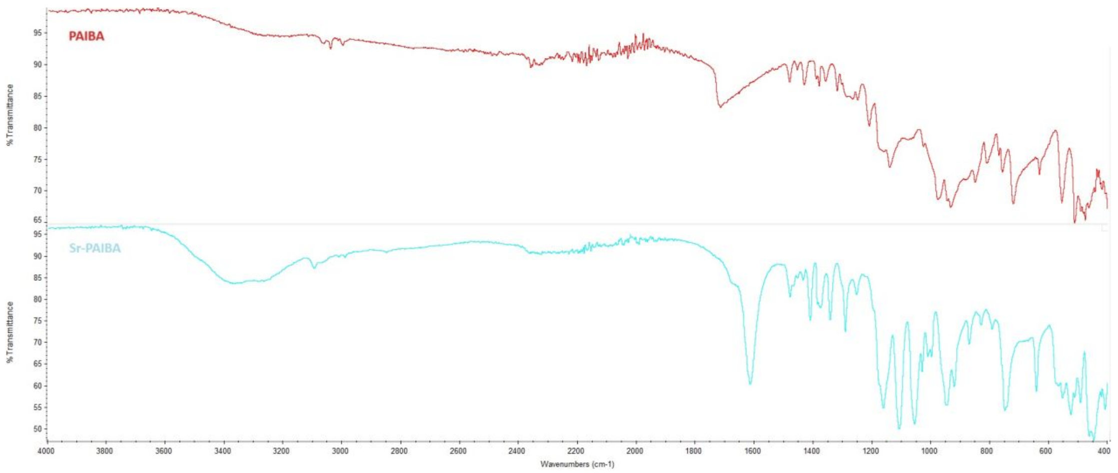
PAIBA
Sr-PAIBA
% Transmittance
% Transmittance
Wavenumbers (cm-1)

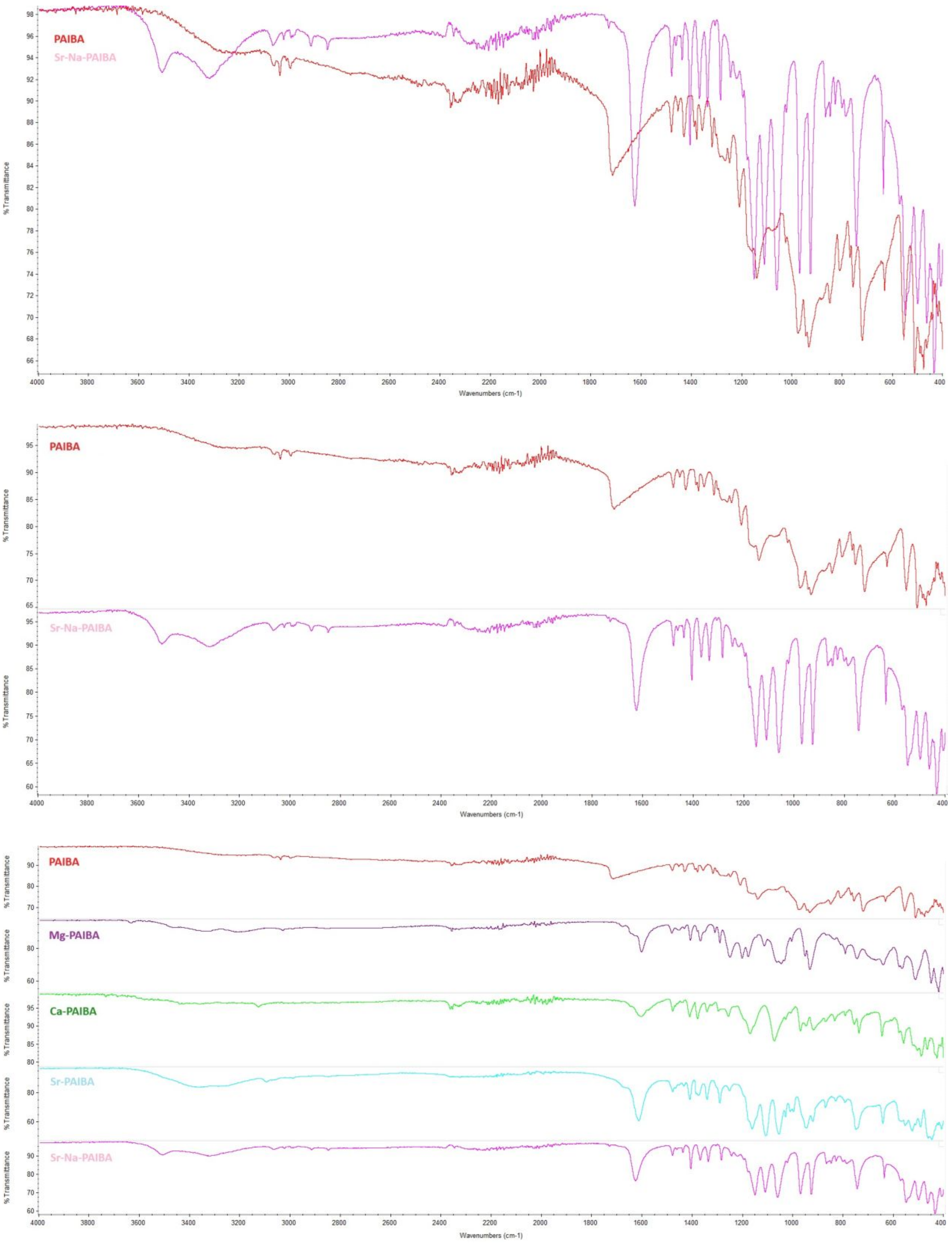

Figure S7. Comparative ATR-IR spectra of M^{2+}-PAIBA compounds (M^{2+} = Mg^{2+}, Ca^{2+}, Sr^{2+}): Overlaid/stacked spectra in comparison with the “free” PAIBA (given in red) and overall comparison of the synthesized compounds.

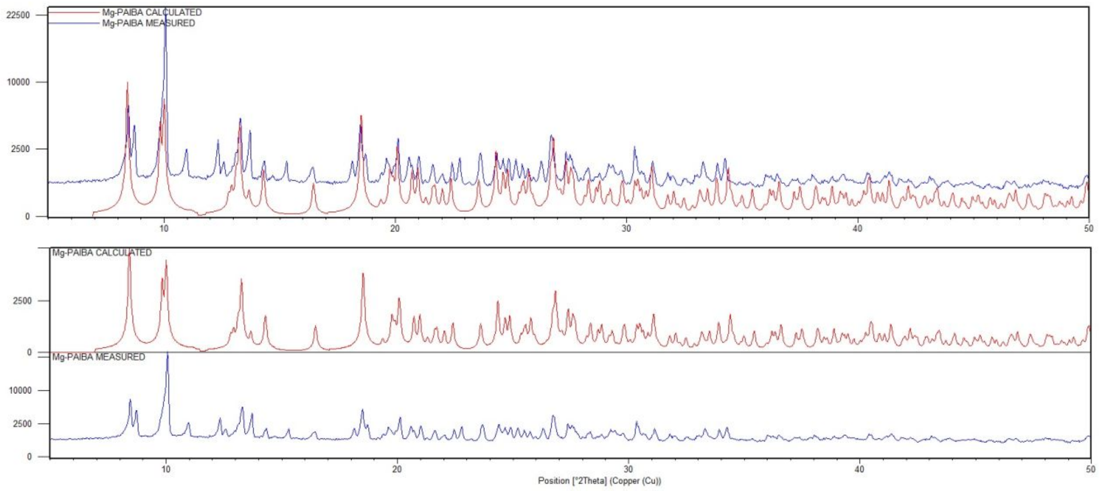

Mg-PAIBA

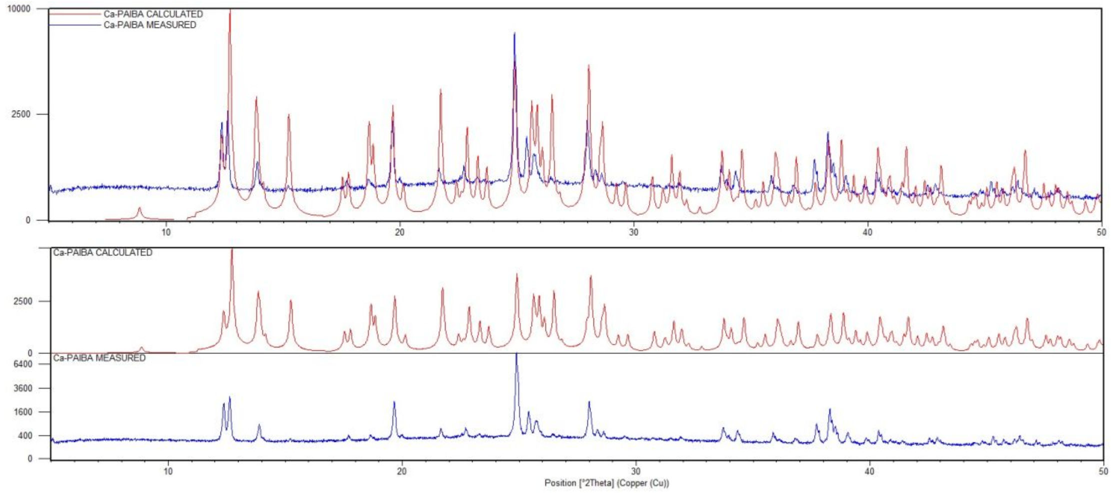

Ca-PAIBA

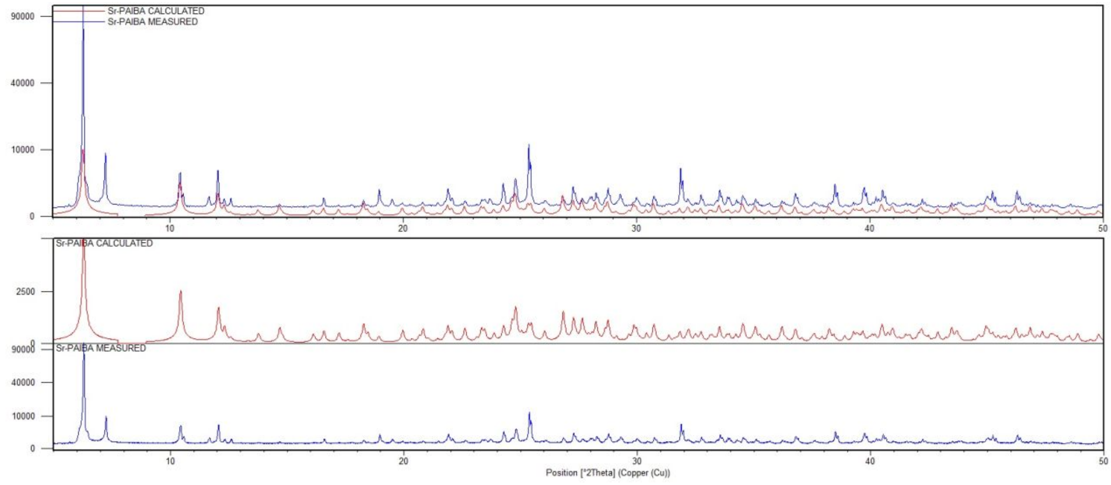

Sr-PAIBA

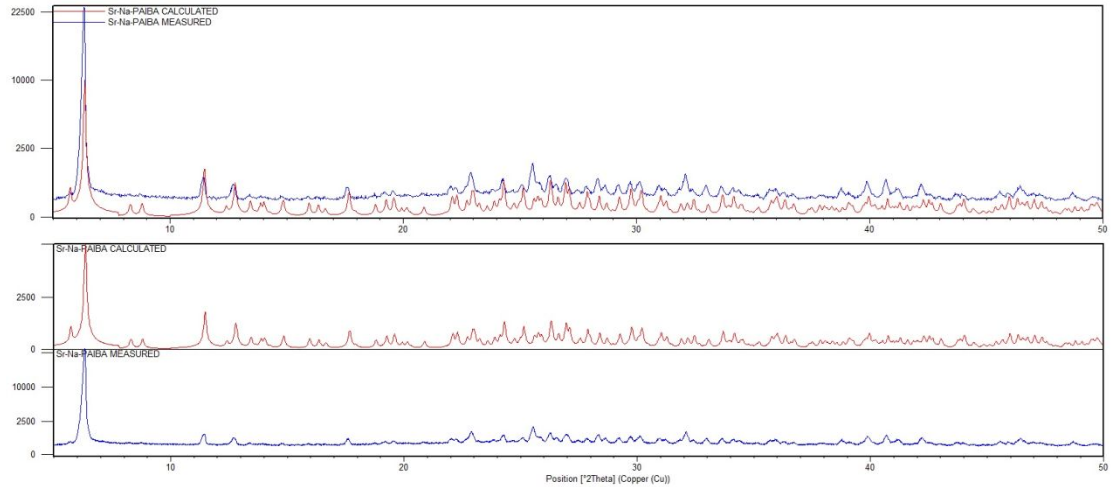

Sr-Na-PAIBA

Figure S8. Overlaid and stacked comparative powder X-ray diffraction diagrams [calculated (red) vs. measured (blue)] of all M^{2+}-PAIBA compounds (M^{2+} = Mg^{2+}, Ca^{2+}, Sr^{2+})

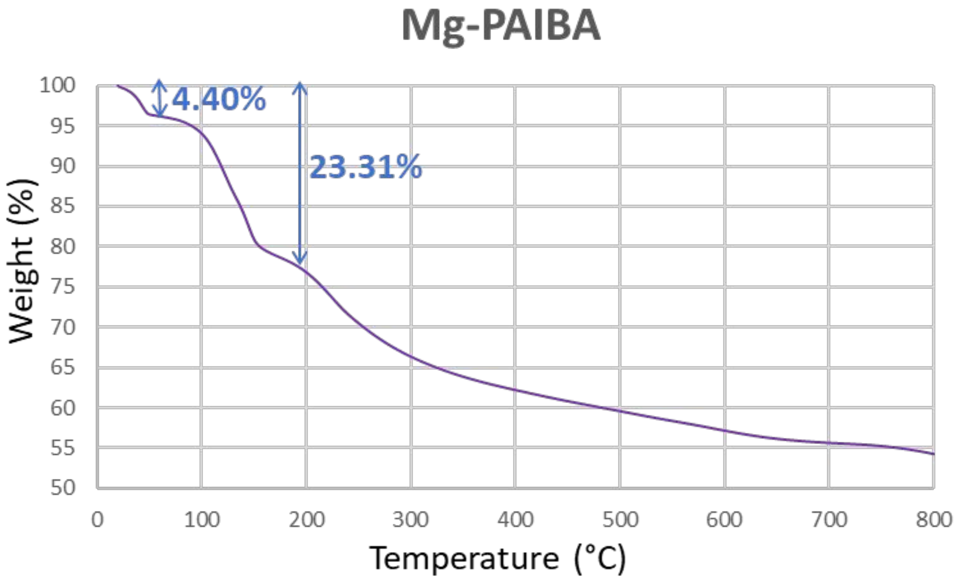

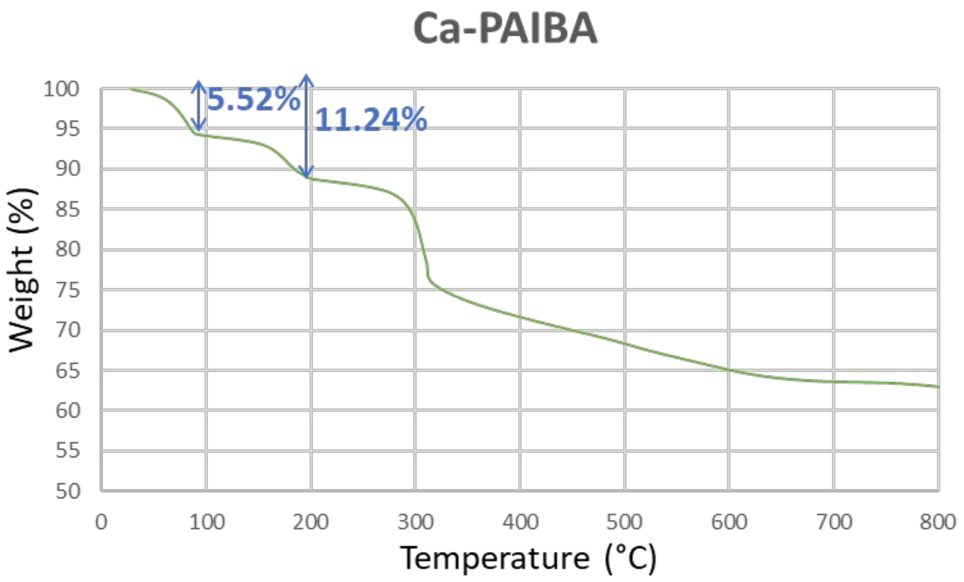
Ca-PAIBA
100
95
90
85
80
75
70
65
60
55
50
Weight (%)
5.52%
11.24%
0
100
200
300
400
500
600
700
800
Temperature (°C)

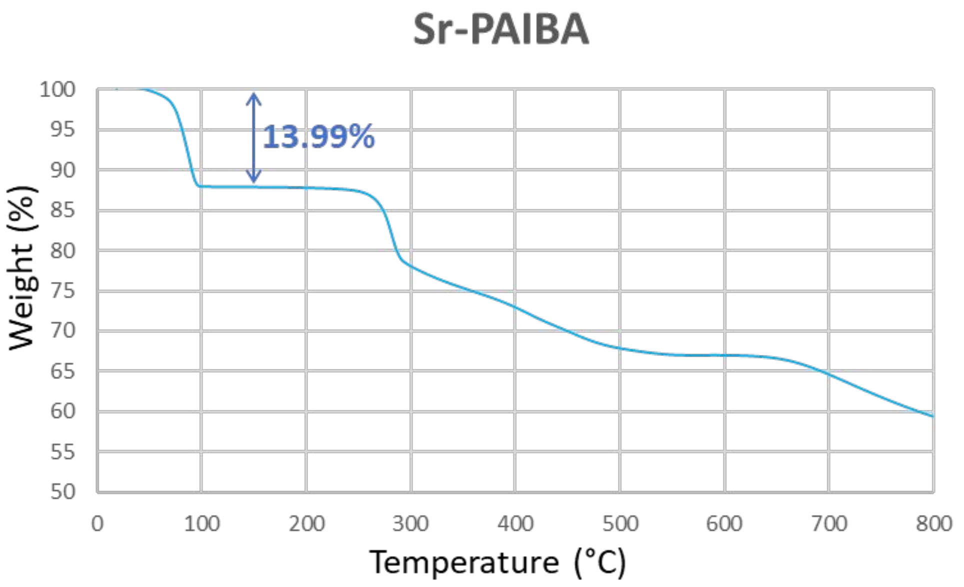
Sr-PAIBA
100
95
90
85
80
75
70
65
60
55
50
Weight (%)
13.99%
0
100
200
300
400
500
600
700
800
Temperature (°C)

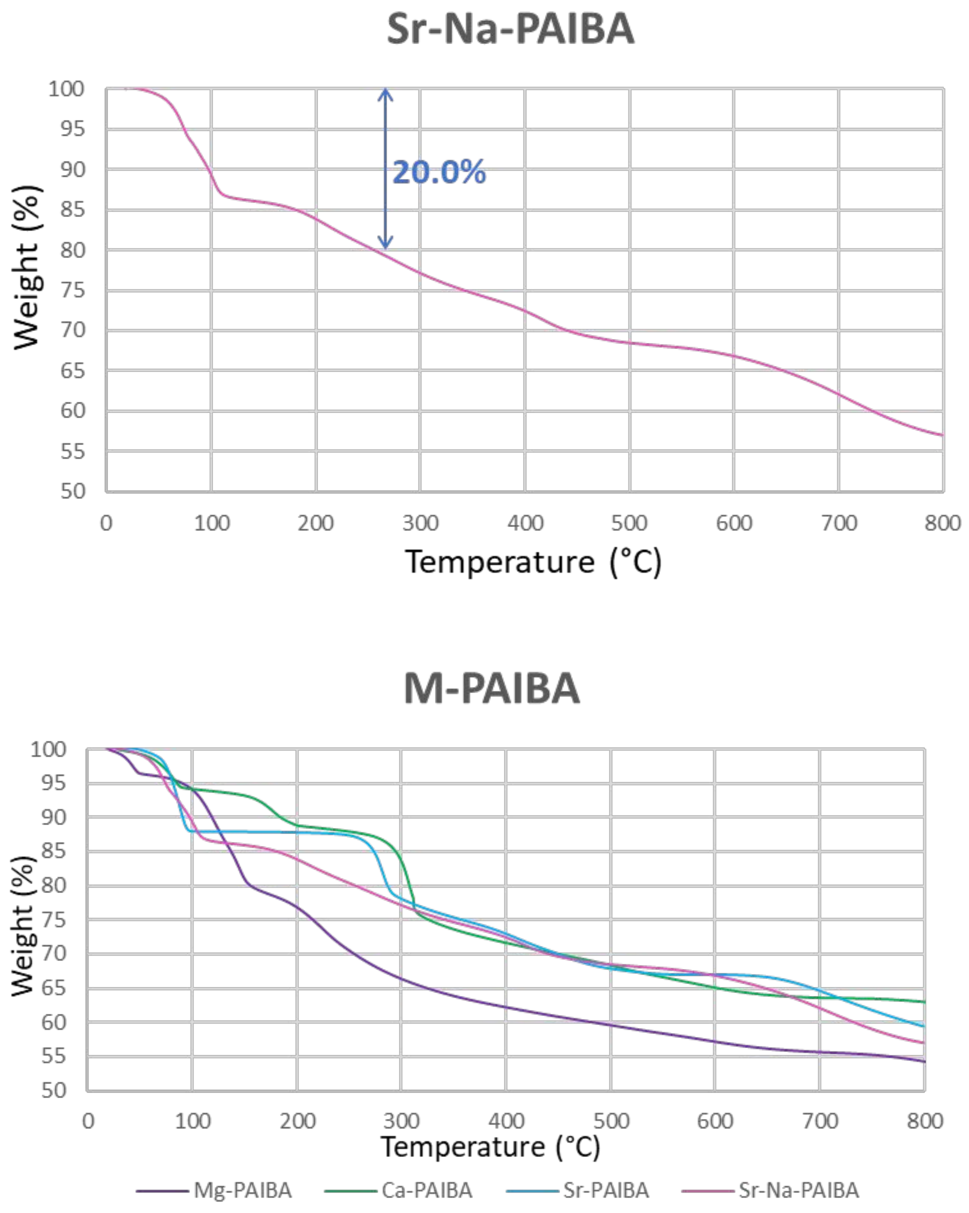

Figure S9. TGA traces of all M^{2+}-PAIBA compounds ($M^{2+} = Mg^{2+}, Ca^{2+}, Sr^{2+}$).

3. Corrosion inhibition experiments

Table S2. Corrosion rates (mm/y) and % inhibition of all systems containing BPMGLY.*

**System**	**Concentration**	**Corrosion rate (mm/y), % inhibition**		
		pH = 4	***pH = 5***	***pH = 6***
BPMGLY	0.1 mM	0.019 mm/y, 78 %	0.049 mm/y, 47 %	0.045 mm/y, 55 %
	0.5 mM	0.032 mm/y, 63 %	0.055 mm/y, 41 %	0.054 mm/y, 46 %
	1.0 mM	0.074 mm/y, 21 %	0.059 mm/y, 44 %	0.056 mm/y, 48 %
BPMGLY - Mg^{2+}	0.1 mM	0.049 mm/y, 43 %	0.053 mm/y, 43 %	0.038 mm/y, 62 %
	0.5 mM	0.041 mm/y, 52 %	0.057 mm/y, 38 %	0.053 mm/y, 47 %
	1.0 mM	0.065 mm/y, 33 %	0.059 mm/y, 44 %	0.051 mm/y, 53 %
BPMGLY - Ca^{2+}	0.1 mM	0.067 mm/y, 22 %	0.049 mm/y, 48 %	0.043 mm/y, 57 %
	0.5 mM	0.053 mm/y, 39 %	0.061 mm/y, 34 %	0.051 mm/y, 49 %
	1.0 mM	0.062 mm/y, 36 %	0.054 mm/y, 49 %	0.046 mm/y, 58 %
BPMGLY - Sr^{2+}	0.1 mM	0.046 mm/y, 47 %	0.061 mm/y, 34 %	0.041 mm/y, 59 %
	0.5 mM	0.054 mm/y, 37 %	0.058 mm/y, 37 %	0.057 mm/y, 43 %
	1.0 mM	0.066 mm/y, 32 %	0.062 mm/y, 41 %	0.040 mm/y, 63 %
BPMGLY - Ba^{2+}	0.1 mM	0.046 mm/y, 47 %	0.054 mm/y, 42 %	0.042 mm/y, 58 %
	0.5 mM	0.046 mm/y, 46 %	0.037 mm/y, 60 %	0.046 mm/y, 53 %
	1.0 mM	0.060 mm/y, 38 %	0.054 mm/y, 49 %	0.036 mm/y, 66 %

* Inhibition efficiencies > 50 % are highlighted in green and those between 0 % and 50 % are in blue.

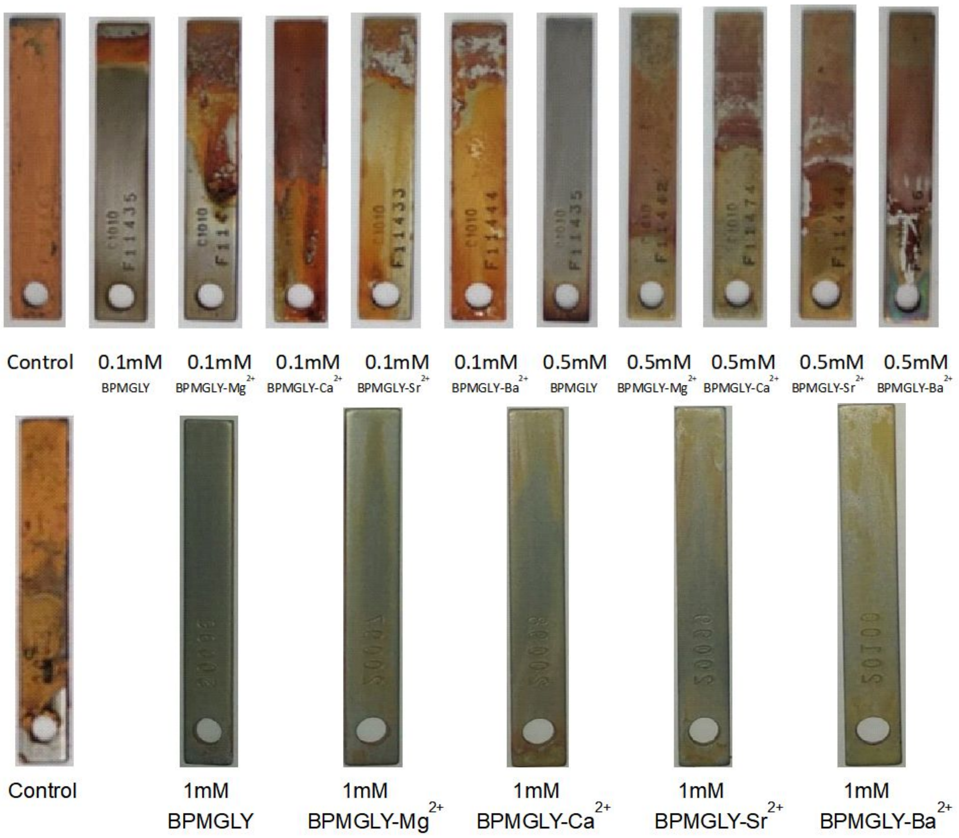

Figure S10. Optical images of the carbon steel surfaces after immersion in the presence of BPMGLY inhibitor and its combination with alkaline earth metal ions, at pH 4.0 and at concentrations 0.1 mM, 0.5 mM and 1.0 mM.

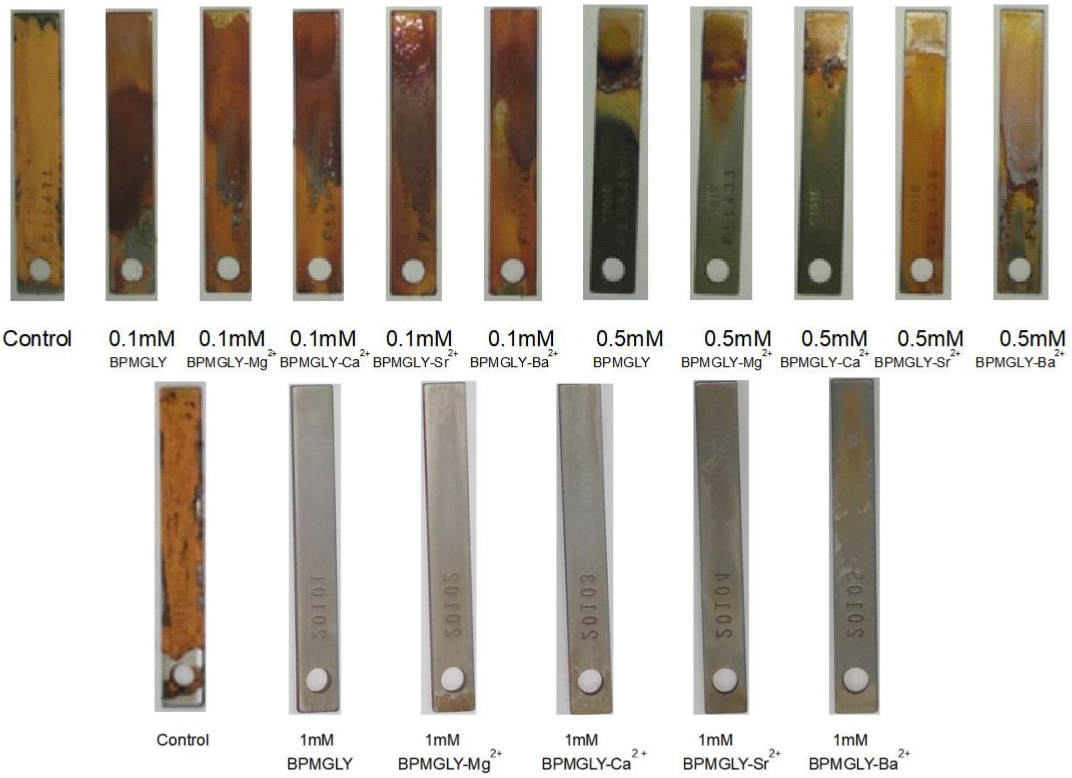

Figure S11. Optical images of the carbon steel surfaces after immersion in the presence of BPMGLY inhibitor and its combination with alkaline earth metal ions, at pH 5.0 and at concentrations 0.1 mM, 0.5 mM and 1.0 mM.

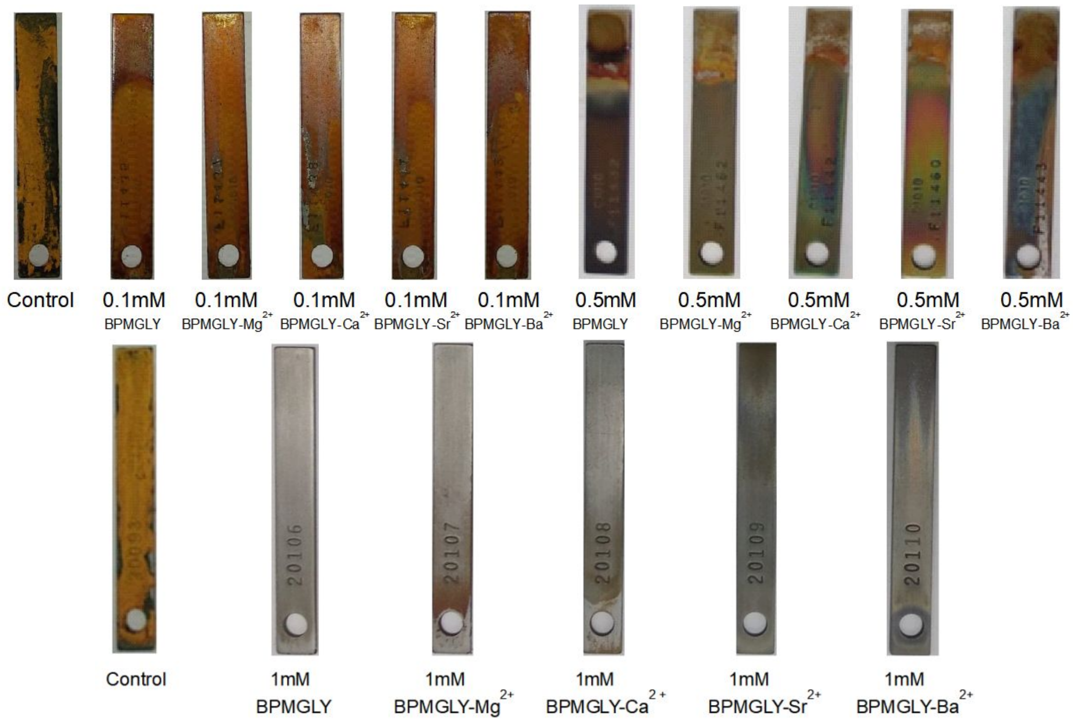

Figure S12. Optical images of the carbon steel surfaces after immersion in the presence of BPMGLY inhibitor and its combination with alkaline earth metal ions, at pH 6.0 and at concentrations 0.1 mM, 0.5 mM and 1.0 mM.

Table S3. Corrosion rates (mm/y) and % inhibition of all systems containing PAIBA.*

**System**	**Concentration**	**Corrosion rate (mm/y), % inhibition**		
		pH = 4	***pH = 5***	***pH = 6***
PAIBA	0.1 mM	0.027 mm/y, 69 %	0.036 mm/y, 62 %	0.051 mm/y, 49 %
	0.5 mM	0.037 mm/y, 57 %	0.032 mm/y, 65 %	0.072 mm/y, 28 %
	1.0 mM	0.063 mm/y, 35 %	0.058 mm/y, 44 %	0.061 mm/y, 43 %
PAIBA - Mg^{2+}	0.1 mM	0.048 mm/y, 45 %	0.046 mm/y, 51 %	0.040 mm/y, 60 %
	0.5 mM	0.045 mm/y, 48 %	0.064 mm/y, 31 %	0.063 mm/y, 37 %
	1.0 mM	0.067 mm/y, 3 %	0.061 mm/y, 42 %	0.062 mm/y, 42 %
PAIBA - Ca^{2+}	0.1 mM	0.054 mm/y, 37 %	0.040 mm/y, 56 %	0.045 mm/y, 55 %
	0.5 mM	0.052 mm/y, 40 %	0.065 mm/y, 29 %	0.062 mm/y, 38 %
	1.0 mM	0.068 mm/y, 30 %	0.062 mm/y, 41 %	0.065 mm/y, 30 %
PAIBA - Sr^{2+}	0.1 mM	0.056 mm/y, 36 %	0.042 mm/y, 54 %	0.043 mm/y, 57 %
	0.5 mM	0.071 mm/y, 18 %	0.059 mm/y, 36 %	0.067 mm/y, 33 %
	1.0 mM	0.068 mm/y, 30 %	0.058 mm/y, 45 %	0.057 mm/y, 47 %
PAIBA - Ba^{2+}	0.1 mM	0.053 mm/y, 39 %	0.109 mm/y, -17% (dissolution of metal)	0.040 mm/y, 60 %
	0.5 mM	0.073 mm/y, 15 %	0.060 mm/y, 35 %	0.064 mm/y, 35 %
	1.0 mM	0.069 mm/y, 29 %	0.064 mm/y, 39 %	0.064 mm/y, 40 %

* Inhibition efficiencies > 50 % are highlighted in green and those between 0 % and 50 % are in blue. Metal dissolution is highlighted in red.

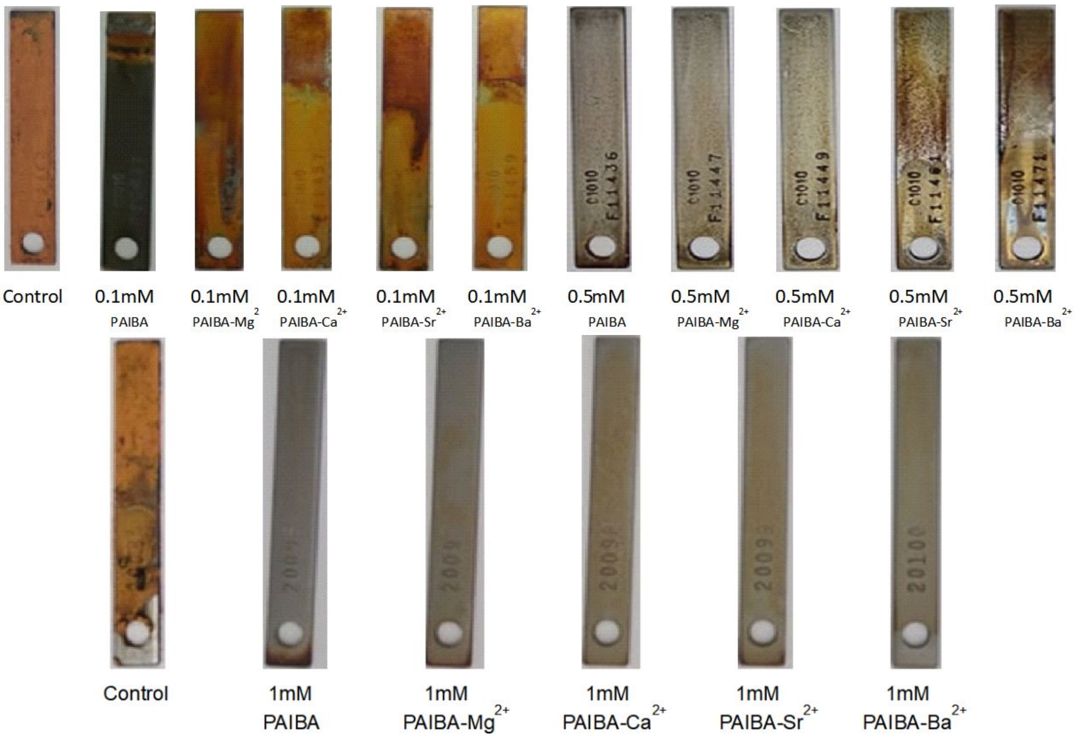

Figure S13. Optical images of the carbon steel surfaces after immersion in the presence of PAIBA inhibitor and its combination with alkaline earth metal ions, at pH 4.0 and at concentrations 0.1 mM, 0.5 mM and 1.0 mM.

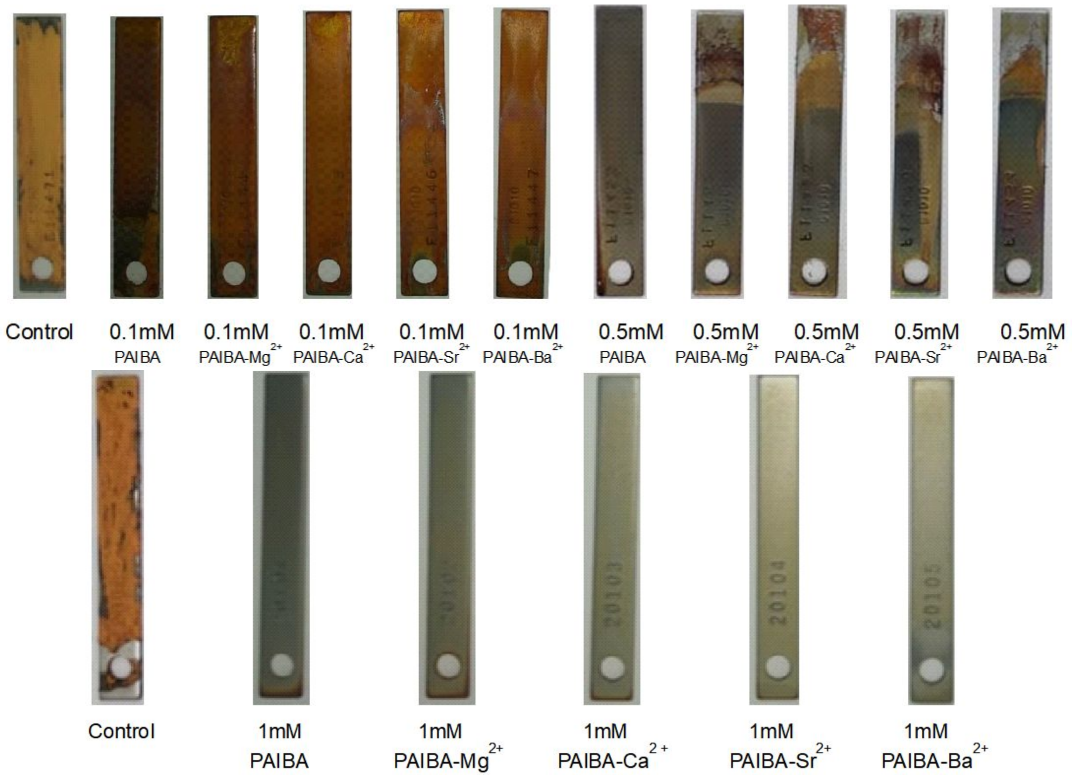

Figure S14. Optical images of the carbon steel surfaces after immersion in the presence of PAIBA inhibitor and its combination with alkaline earth metal ions, at pH 5.0 and at concentrations 0.1 mM, 0.5 mM and 1.0 mM.

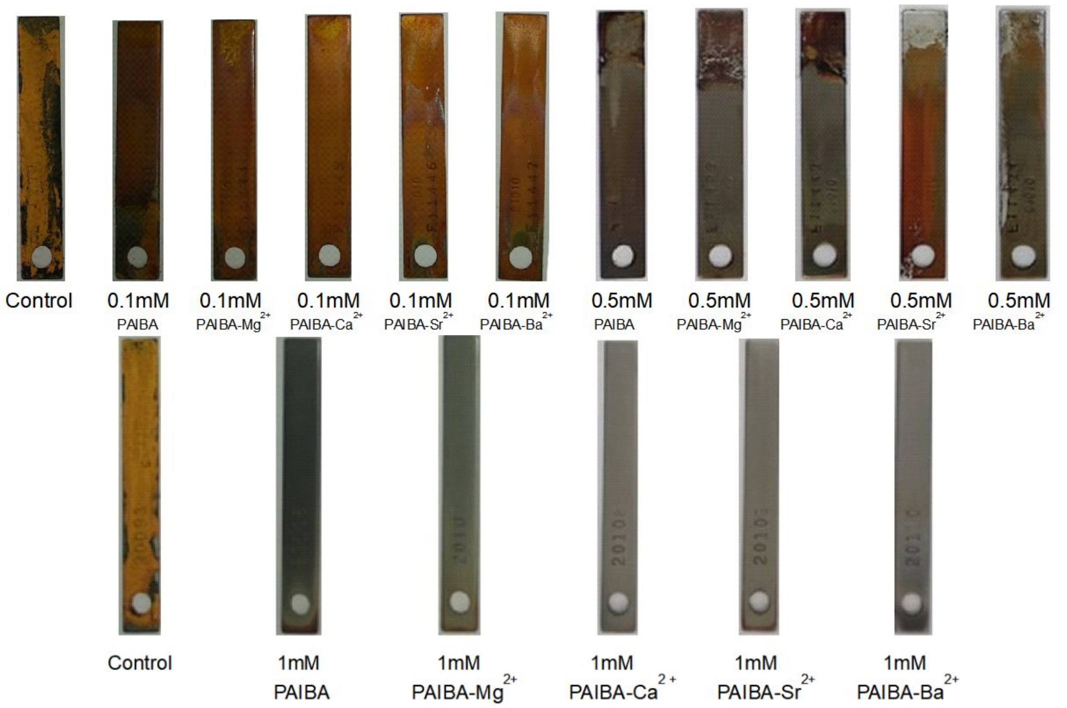

Figure S15. Optical images of the carbon steel surfaces after immersion in the presence of PAIBA inhibitor and its combination with alkaline earth metal ions, at pH 6.0 and at concentrations 0.1 mM, 0.5 mM and 1.0 mM.

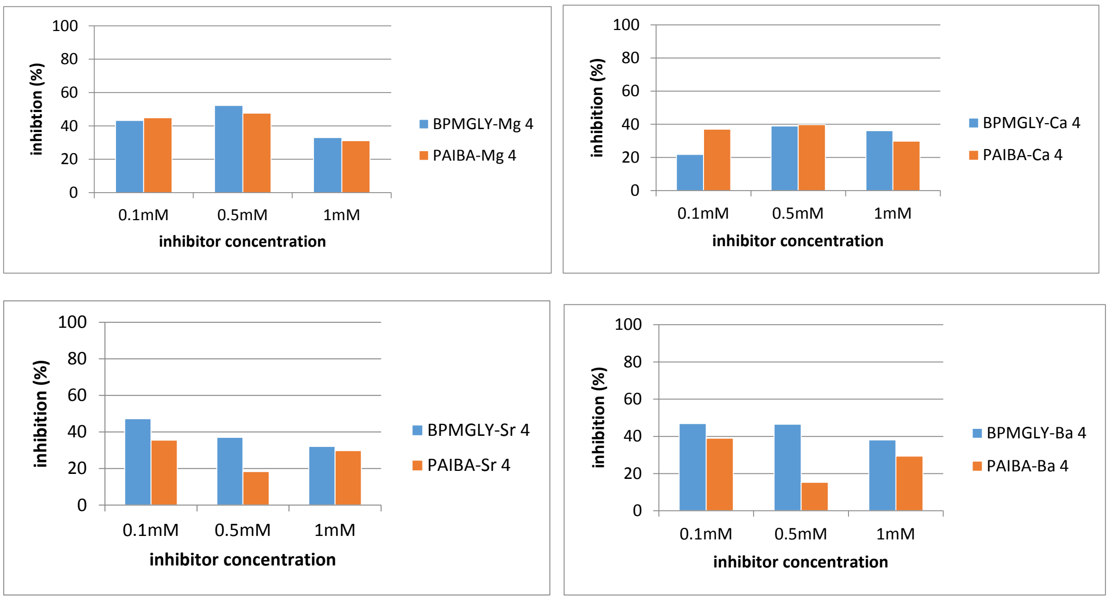

Figure S16. Comparative inhibition efficiency (%) data for the metal-PAIBA and metal-BPMGLY inhibitor systems, at pH = 4.0 and at concentrations 0.1 mM, 0.5 mM and 1.0 mM.

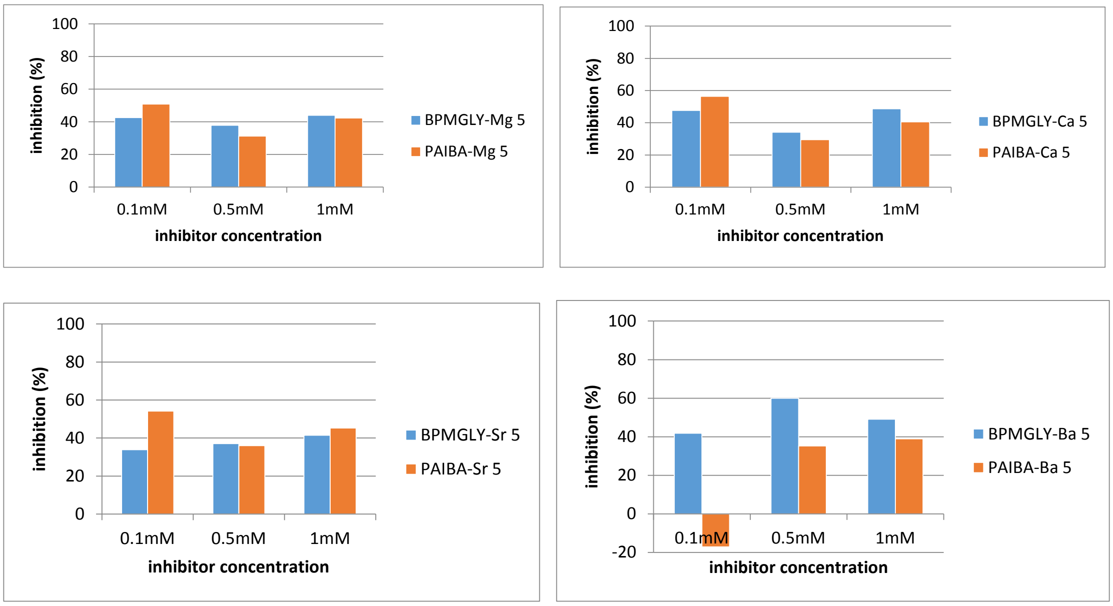

Figure S17. Comparative inhibition efficiency (%) data for the metal-PAIBA and metal-BPMGLY inhibitor systems, at pH = 5.0 and at concentrations 0.1 mM, 0.5 mM and 1.0 mM.

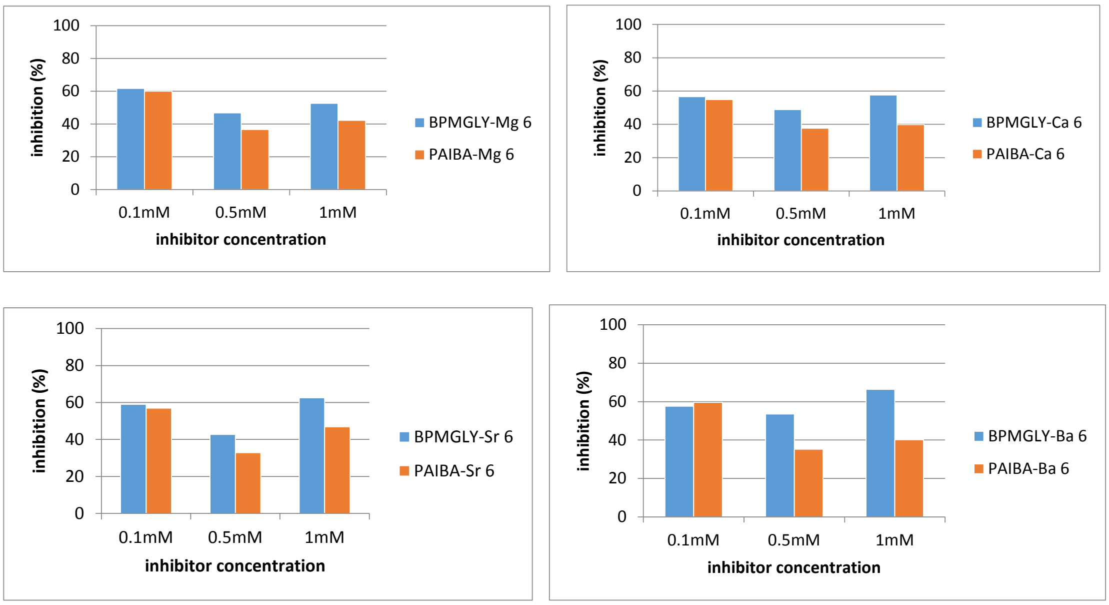

Figure S18. Comparative inhibition efficiency (%) data for the metal-PAIBA and metal-BPMGLY inhibitor systems, at pH = 6.0 and at concentrations 0.1 mM, 0.5 mM and 1.0 mM.

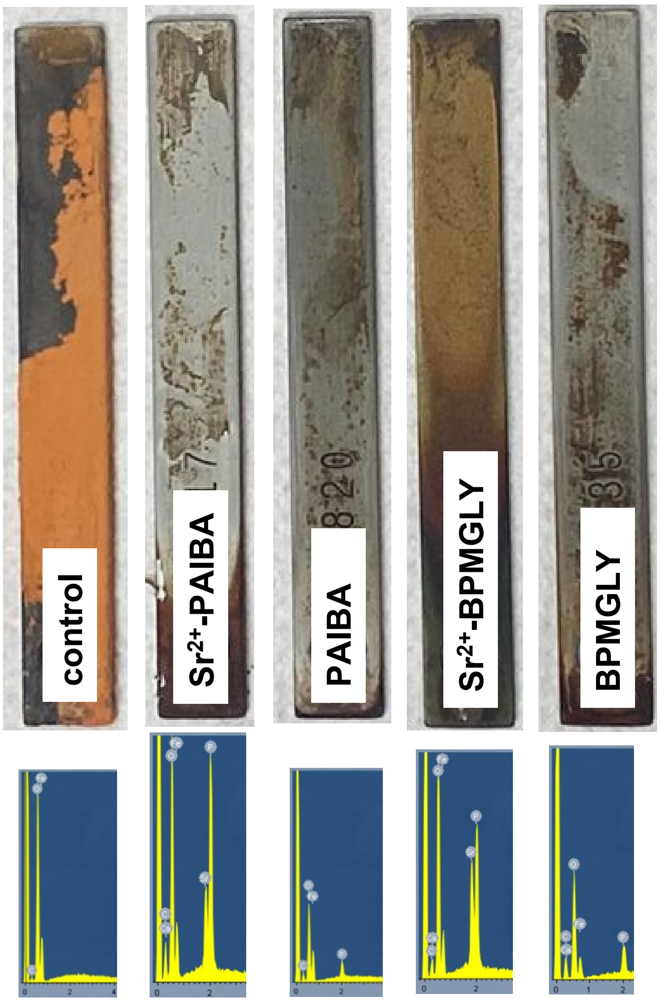

Figure S19. Optical images of the carbon steel surfaces used for the EDS studies, after immersion for ~ 10 days in the absence of inhibitors (control), and in the presence of inhibitor as shown, at pH 6.0 at the concentration 1.0 mM of PAIBA, BPMGLY, and Sr^{2+}. All specimens show the expected presence of Fe. Specimens treated only with phosphonate show the presence of P, whereas those treated with Sr^{2+} and phosphonate show the presence of both Sr and P.
